# Cannabinoid CB_1_ receptor gene inactivation in oligodendrocyte precursors disrupts oligodendrogenesis and myelination in mice

**DOI:** 10.1038/s41419-022-05032-z

**Published:** 2022-07-07

**Authors:** Aníbal Sánchez-de la Torre, Tania Aguado, Alba Huerga-Gómez, Silvia Santamaría, Antonietta Gentile, Juan Carlos Chara, Carlos Matute, Krisztina Monory, Susana Mato, Manuel Guzmán, Beat Lutz, Ismael Galve-Roperh, Javier Palazuelos

**Affiliations:** 1 Instituto Ramón y Cajal de Investigación Sanitaria (IRYCIS), Madrid, Spain; 2 Centro de Investigación Biomédica en Red sobre Enfermedades Neurodegenerativas (CIBERNED), Madrid, Spain; 3 Department of Biochemistry and Molecular Biology, Complutense University, Instituto Universitario de Investigación en Neuroquímica (IUIN), Madrid, Spain; 4 Department of Biomedicine and Prevention, Tor Vergata University, Rome, Italy; 5 University Medical Center, Institute of Physiological Chemistry, Mainz, Germany; 6 Department of Neurosciences, University of the Basque Country UPV/EHU, Leioa, Spain; 7 Achucarro Basque Center for Neuroscience, Leioa, Spain; 8 Biocruces, Bizkaia, Spain

**Keywords:** Gliogenesis, Differentiation

## Abstract

Cannabinoids are known to modulate oligodendrogenesis and developmental CNS myelination. However, the cell-autonomous action of these compounds on oligodendroglial cells in vivo, and the molecular mechanisms underlying these effects have not yet been studied. Here, by using oligodendroglial precursor cell (OPC)-targeted genetic mouse models, we show that cannabinoid CB_1_ receptors exert an essential role in modulating OPC differentiation at the critical periods of postnatal myelination. We found that selective genetic inactivation of CB_1_ receptors in OPCs in vivo perturbs oligodendrogenesis and postnatal myelination by altering the RhoA/ROCK signaling pathway, leading to hypomyelination, and motor and cognitive alterations in young adult mice. Conversely, pharmacological CB_1_ receptor activation, by inducing E3 ubiquitin ligase-dependent RhoA proteasomal degradation, promotes oligodendrocyte development and CNS myelination in OPCs, an effect that was not evident in OPC-specific CB_1_ receptor-deficient mice. Moreover, pharmacological inactivation of ROCK in vivo overcomes the defects in oligodendrogenesis and CNS myelination, and behavioral alterations found in OPC-specific CB_1_ receptor-deficient mice. Overall, this study supports a cell-autonomous role for CB_1_ receptors in modulating oligodendrogenesis in vivo, which may have a profound impact on the scientific knowledge and therapeutic manipulation of CNS myelination by cannabinoids.

## Introduction

During developmental CNS myelination, oligodendrocyte progenitor cells (OPCs) proliferate, migrate, and differentiate into mature myelinating oligodendrocytes (OLs), which generate the myelin sheath internode and, thereby, interact with axons to organize the nodal, paranodal, and juxtaparanodal regions [1, 2]. Thus, the OL developmental program is temporally and spatially controlled by a high number of extracellular signals that coordinately regulate essential intracellular signaling pathways and their downstream transcriptional programs. Alterations in essential genes modulating OPC differentiation and/or OL maturation cause myelination defects, which is translated into neuronal dysfunction and, eventually, behavioral alterations in mice [2–5]. Despite the identification of a high number of these regulatory signals, the whole molecular network that controls developmental oligodendrogenesis and CNS myelination has remained incomplete.

Almost two decades after the first evidence supporting that cannabinoid compounds modulate OL development and CNS myelination [6, 7], several pharmacological studies have shown that synthetic cannabinoids [8], phytocannabinoids [9], and endocannabinoids (eCBs) [10] modulate oligodendrogenesis and CNS myelination. Elevated levels of the endocannabinoid 2-arachidonoylglycerol (2-AG) upon pharmacological inhibition of the 2-AG-degrading enzyme monoacylglycerol lipase (MAGL) enhances OL development in cultured OPCs [11], both at embryonic stages in mice [10] and in the Theiler’s murine encephalomyelitis virus (TMEV) progressive mouse model of multiple sclerosis (MS) [12, 13]. Moreover, administration of the phytocannabinoid Δ^9^-tetrahydrocannabinol (THC) [9] or of synthetic cannabinoids [8] promotes OPC differentiation and developmental CNS myelination, as well as OL regeneration and functional CNS remyelination upon cuprizone-induced demyelination [14]. Moreover, the phytocannabinoid cannabidiol (CBD) prevents hypoxia/ischemia-induced hypomyelination in newborn rats [15]. Of note, collectively, these studies have only been based on systemic pharmacological approaches. Owing to the abundant expression of CB_1_ receptors in various neuronal and glial cell populations [16], the cell-autonomous action of cannabinoid compounds in OPCs to modulate OL development in vivo has remained unexplored. Thus, deciphering the cellular neurobiology of the eCB system may help to identify the cellular targets of cannabinoids under physiological or pathophysiological settings.

Here, by using genetic mouse models aimed to inactivate CB_1_ receptor gene expression selectively in OPCs, we show that CB_1_ receptors exert an essential function in modulating OPC differentiation and oligodendrogenesis during postnatal myelination in vivo. We found that selective depletion of CB_1_ receptor signaling in OPCs, by altering the RhoA/ROCK signaling pathway, prevents cell differentiation, perturbs oligodendrogenesis and postnatal myelination, and causes hypomyelination as well as motor and cognitive defects at young adult ages. Moreover, pharmacological inactivation of ROCK in vivo overcomes the defects in oligodendrogenesis and functional CNS myelination of CB_1_ receptor deficient OPCs. This study supports an essential role for CB_1_ receptors in modulating OPC functions and CNS myelination, which may contribute to understanding the complex molecular network that controls CNS myelination.

## Results

### Selective CB_1_ receptor gene inactivation in OPCs in vivo

To interrogate a cell-autonomous role for CB_1_ receptors in the modulation of oligodendrogenesis and CNS myelination in vivo, we generated a new mouse line by crossing the CB_1_^f/f^ [17] mouse line with the *Ng2*-CreERT2 [18] and Rosa-stop-Ai6 [19] mouse lines. This would conceivably deplete CB_1_ receptors gene (*Cnr*_*1*_) expression selectively in OPCs upon tamoxifen (TAM) administration, and ZsGreen1 fluorescently label OPCs to track OL differentiation along the process of CNS myelination (Fig. S1A). We found approximately 77.7% of recombination efficiency in *Ng2*/Ai6-CB_1_KO and *Ng2*/Ai6-CB_1_HET mice by quantifying the percentage of oligodendroglial-lineage Olig2^+^ cells that expressed the Ai6 recombinant transgene by immunofluorescence analysis in the developing *corpus callosum* (CC) (Fig. S1B). We verified the recombination in the CB_1_ receptor locus by genomic DNA analysis of *Ng2*/Ai6-CB_1_KO and *Ng2*/Ai6-CB_1_HET CC FAC-sorted cells at postnatal day 10 (P10) (Fig. S1C, D) [20]. We also confirmed CB_1_ protein depletion in *Ng2/*Ai6-CB_1_KO CC isolated cells at P10 by immunofluorescence (Fig. S1E).

### CB_1_ receptor gene inactivation in OPCs disrupts postnatal oligodendrogenesis

**Fig. 1:**
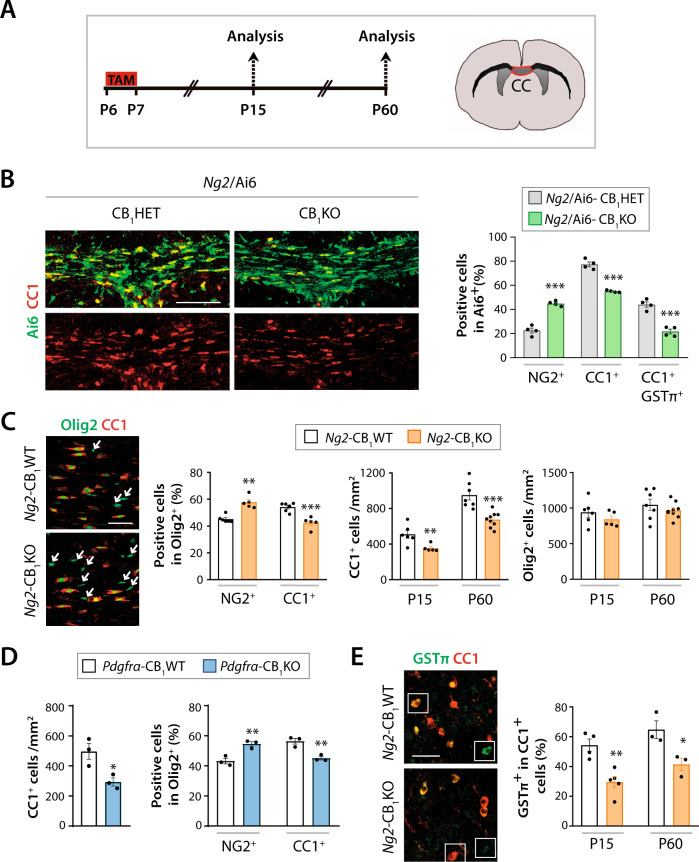
CB_1_ receptor gene inactivation in OPCs disrupts postnatal oligodendrogenesis. **A** Timeline of tamoxifen administrations and time points of analysis. Tamoxifen-driven recombination was induced at postnatal day 6 (P6-P7) in **B** *Ng2*/Ai6-CB_1_KO, **C**, **E** *Ng2*-CB_1_KO, **D** *Pdgfrα*-CB_1_KO and control mice, and oligodendrogenesis was analyzed in the *corpus callosum* at P15 and P60. Immunofluorescence analysis and quantification of **B** the percentage of NG2^+^ OPCs, CC1^+^ OLs and GSTπ^+^CC1^+^ mature OLs among the recombinant Rosa-Ai6^+^ population at P15, (**C**, left panel, **D**) the percentage of NG2^+^ OPCs, CC1^+^ OLs among Olig2^+^ cells at P15, (**C, D**) Olig2^+^ and CC1^+^ OL cell densities at P15 **C**, **D** and P60 **C**, and **E** the percentage of mature GSTπ^+^ OLs among CC1^+^ OLs at P15 and P60. Arrows in **B** indicate Olig2^+^CC1^neg^ cells. Data are shown as mean + /− SEM. *n* = 4 for **B**, *n* = 5–9 for **C**, *n* = 3 for **D**, *n* = 3–5 for **E** independent data points used per experimental group. *p ≤ 0.05, **p ≤ 0.01, ***p ≤ 0.001 vs *Ng2*/Ai6-CB_1_HET, *Ng2*-CB_1_WT or *Pdgfrα*-CB_1_WT mice, by two-tailed unpaired Student’s t-test for **B**, **D**, **E**, and by two-tailed unpaired Student’s t-test or Mann Whitney test for **C**. Scale bars, 80 µm for **B**, and 30 µm for **C** and **E**’.

To study the role for CB_1_ receptors in oligodendrogenesis, we induced TAM-driven recombination in *Ng2*/Ai6-CB_1_KO and *Ng2*/Ai6-CB_1_HET mice at P6 and P7 and performed a differentiation-state analysis of the OL-lineage populations by immunofluorescence in the CC at P15 and at a young adult age (P60) (Fig. 1A). Analysis revealed a higher proportion of NG2^+^ OPCs and a reduced proportion of CC1^+^ OLs within the recombinant Ai6^*+*^ population in the CC of *Ng2*/Ai6-CB_1_KO mice compared to their controls (*Ng2*/Ai6-CB_1_HET), thus pointing to a blockade of OPC differentiation (Fig. 1B and Fig. S2A). Similar data were obtained when analyzing the Olig2^+^ cell population in the *Ng2*-CB_1_KO mouse line at P15 (Fig. 1C and Fig. S2B). *Ng2*-CB_1_KO mice also showed a reduced density of CC1^+^ OLs compared to their respective controls (*Ng2*-CB_1_WT mice) at both ages (Fig. 1C). We did not find differences in Olig2^+^ cell densities between *Ng2*-CB_1_KO mice and their control *Ng2*-CB_1_WT littermates at both ages (Fig. 1C), indicating that CB_1_ receptor gene inactivation in OPCs disrupts cell differentiation without affecting OPC cell survival. We provided further support of these data by generating another OPC-specific mouse line upon crossing *Pdgfrα*-Cre [21] and CB_1_^f/f^ animals. *Pdgfrα*-CB_1_KO mice also showed a reduced CC1^+^ OL cell density as well as a reduced proportion of CC1^+^ cells among Olig2^+^ cells in the developing CC compared to their control *Pdgfrα*-CB_1_WT mice at P15 (Fig. 1D and Fig. S2C). Taken together, these results indicate that CB_1_ receptors regulate OPC differentiation during postnatal development in a cell autonomous manner.

Next, we studied the maturation state of the OL population in the CC of OPC-CB_1_ receptor-deficient mice. Immunofluorescence analysis of OL (CC1) and mature myelinating OL markers (glutathione S-transferase P, GSTπ; myelin-associated glycoprotein MAG) showed a reduced density of GSTπ^+^CC1^+^ or MAG^+^ mature myelinating OLs (Fig. S3A, B), together with a reduced proportion of myelinating GSTπ^+^ OLs within CC1^+^ OLs cells, in *Ng2*-CB_1_KO mice compared to their *Ng2*-CB_1_WT control mice at P15 and P60 (Fig. 1E). We also found a reduced proportion of CC1^+^GSTπ^+^ cells within the Ai6^+^ population in the CC of *Ng2*/Ai6-CB_1_KO mice compared to their *Ng2*/Ai6-CB_1_HET controls (Fig. 1B and Fig. S3C), thus suggesting that CB_1_ receptors modulate not only OPC differentiation but also OL maturation. Altogether, these findings indicate that CB_1_ receptor ablation in OPCs prevents cell differentiation and disrupts oligodendrogenesis during the critical period of postnatal myelination.

### CB_1_ receptor gene inactivation in OPCs disrupts postnatal CNS myelination

**Fig. 2:**
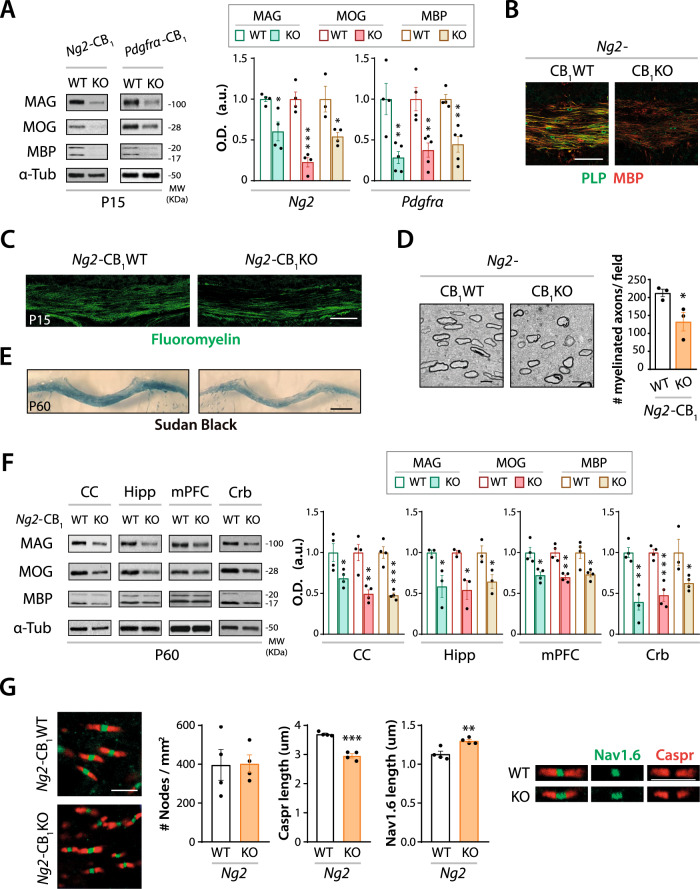
Genetic inactivation of CB_1_ receptor in OPCs disrupts developmental myelination. Tamoxifen-driven recombination was induced in *Ng2*-CB_1_KO, *Pdgfrα*-CB_1_KO and control mice at postnatal day 6 (P6-P7) and tissue was analyzed in the *corpus callosum* (CC) at P15 and P60. **A** and **F** Western blot analysis of myelin protein levels, such as myelin-associated glycoprotein (MAG), myelin oligodendrocyte glycoprotein (MOG), and myelin basic protein (MBP) from CC extracts at P15 (**A**) or P60 (**F**), or cerebellar (Crb), hippocampal (Hipp), and medial prefrontal cortex (mPFC) extracts at P60 (**F**). Quantification of optical density (O.D.). **B** Immunofluorescence analysis of proteolipid protein (PLP) and MBP in *Ng2*-CB_1_KO and *Ng2*-CB_1_WT mice at P15. **C** Fluoromyelin staining in the CC at P15. **D** Electron microscopy analysis and quantification of myelinated axon density in *Ng2*-CB_1_KO and *Ng2*-CB_1_WT mice at P15. **E** Representative images of Sudan black staining in the CC of *Ng2*-CB_1_KO and *Ng2*-CB_1_WT mice at P60. **G** Immunofluorescence analysis and quantification of node density and paranode (Caspr) and node (Nav1.6) length in the CC of *Ng2*-CB_1_KO and *Ng2*-CB_1_WT at P60. Data are shown as mean ± SEM. *n* = 3–5 for **A**, *n* = 3 for **D**, **E**, *n* = 3–4 for **E**, **F**, and *n* = 4 for **G**, independent data points used per each experimental group. *p ≤ 0.05, **p ≤ 0.01, ***p ≤ 0.001 vs *Ng2*-CB_1_WT mice, by two-tailed unpaired Student’s t-test for **A**, **D**, **E**, **F** and **G**. Scale bars, 80 µm for **B**, 100 µm for **C**, 4 µm for **D**, 600 µm for **F**, and 3 µm for **G**.

To address whether the observed defects in oligodendrogenesis shown by *Ng2*-CB_1_KO and *Pdgfrα*-CB_1_KO mice impact the myelination process, we analyzed myelination in the CC of these mice and their respective controls at P15. Western blot analysis of dissected CC extracts revealed reduced levels of myelin-associated proteins, such as MAG, myelin oligodendrocyte glycoprotein (MOG), and myelin basic protein (MBP) from *Ng2*-CB_1_KO and *Pdgfrα*-CB_1_KO mice compared to their controls (*Ng2*-CB_1_WT and *Pdgfrα*-CB_1_WT mice respectively) (Fig. 2A). Fluoromyelin staining, as well as MBP and proteolipid protein (PLP) immunofluorescence analysis in the developing CC, supported the hypomyelinated phenotype of *Ng2*-CB_1_KO mice compared to their respective controls (Fig. 2B, C). Moreover, ultrastructural analysis by electron microscopy showed a reduced density of myelinated axons in CC of *Ng2*-CB_1_KO mice compared to their controls (Fig. 2D). These results indicate that CB_1_ receptor deficiency in OPCs at early postnatal ages disrupts developmental CNS myelination.

### CB_1_ receptor gene inactivation in OPCs causes hypomyelination in young adult mice

We next studied whether CB_1_ receptor deficiency in OPCs at early postnatal ages leads to alterations of myelin levels at P60. Tamoxifen-driven recombination was induced at P6 in *Ng2*-CB_1_KO and their controls, and myelination was evaluated in the CC at P60. Sudan black staining unveiled a hypomyelinated CC in adult *Ng2*-CB_1_KO mice compared to controls (Fig. 2E and Fig. S4B). Western blot and real time-PCR analyses revealed reduced protein and mRNA levels of myelin-associated proteins, such as MAG, MOG and MBP, in CC extracts from *Ng2*-CB_1_KO mice compared to their controls (Fig. 2F and Fig. S4A). Furthermore, we also found reduced myelin-related protein levels in other CNS areas of *Ng2*-CB_1_KO mice, such as the cerebellum, the hippocampus, and the medial prefrontal cortex, compared to *Ng2*-CB_1_WT mice (Fig. 2F). Then, we analyzed whether the hypomyelinated phenotype observed upon OPC-CB_1_ receptor depletion impacts node/paranode density or structure. Immunofluorescence analysis of nodal (Nav1.6) and paranodal (Caspr) markers in the CC at P60 revealed an altered nodal and paranodal length, with equal node density in *Ng2*-CB_1_KO mice compared to their controls (Fig. 2G), confirming a defect in myelination in *Ng2*-CB_1_KO mice.

### CB_1_ receptor gene inactivation in OPCs leads to motor and cognitive defects in young adult mice

**Fig. 3:**
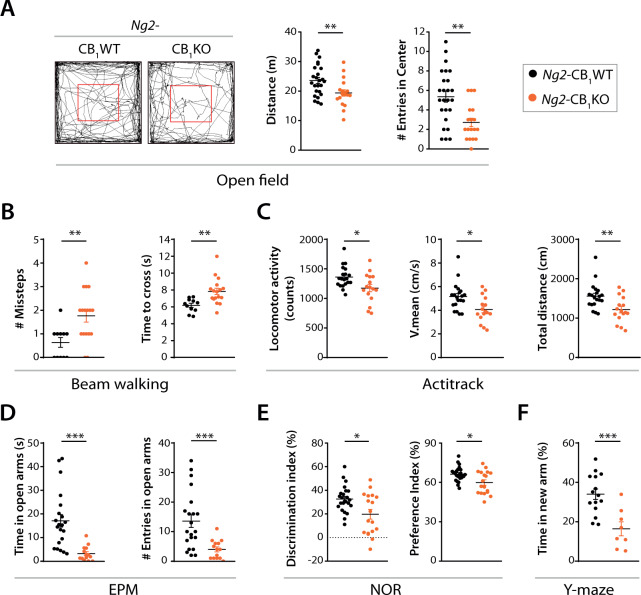
Genetic inactivation of CB_1_ receptor in OPCs causes motor and cognitive defects in young adult mice. Tamoxifen-driven recombination was induced in *Ng2*-CB_1_KO, and control mice at postnatal day 6 (P6-P7) and a battery of behavioural tests was performed at P60. **A** *Open field* test. Representative mouse trajectories, quantification of total distance travelled and the number of entries into the center of the arena. **B** *Beam walking* test. Quantification of the number of missteps and the time spent to cross the beam. **C** *Actitrack* test. Quantification of locomotor activity, mean velocity, and the total distance travelled. **D** *Elevated plus-maze* (EPM) test. Quantification of the time spent in open arms and of the number of entries into open arms. **E** *Novel object recognition* (NOR) test. Quantification of the discrimination index and the preference index. **F** *Y-maze* test. Quantification of the time spent in the new arm. Data are shown as mean ± SEM. *n* = 8–25 independent data points used per each experimental group. *p ≤ 0.05, **p ≤ 0.01, ***p ≤ 0.001 vs *Ng2*-CB_1_WT mice, by two-tailed unpaired Student’s *t*-test, two-tailed unpaired Student’s t-test with Welch’s correction or Man Whitney test.

To address whether the hypomyelinated phenotype observed in *Ng2*-CB_1_KO mice is associated with behavioral alterations, we performed a battery of tests at P60. *Ng2*-CB_1_KO mice showed reduced motor activity and coordination in the *open field* (Fig. 3A), *beam walking* (Fig. 3B), and *Actitrack* (Fig. 3C) tests compared to *Ng2*-CB_1_WT mice. *Ng2*-CB_1_KO also showed an anxiety-like behavior in the *open field* and *elevated plus-maze* (EPM) (Fig. 3A, D) tests, and memory deficits in the *novel object recognition* (NOR, Fig. 3E) and *y-maze* (Fig. 3F) tests. Altogether, these observations show that CB_1_ receptor deficiency in OPCs at early postnatal ages causes hypomyelination at young adult ages, which is translated into impaired motor and memory functions, and anxiety-like behaviors.

### CB_1_ receptors modulate OPC differentiation through the RhoA/ROCK signaling axis

**Fig. 4:**
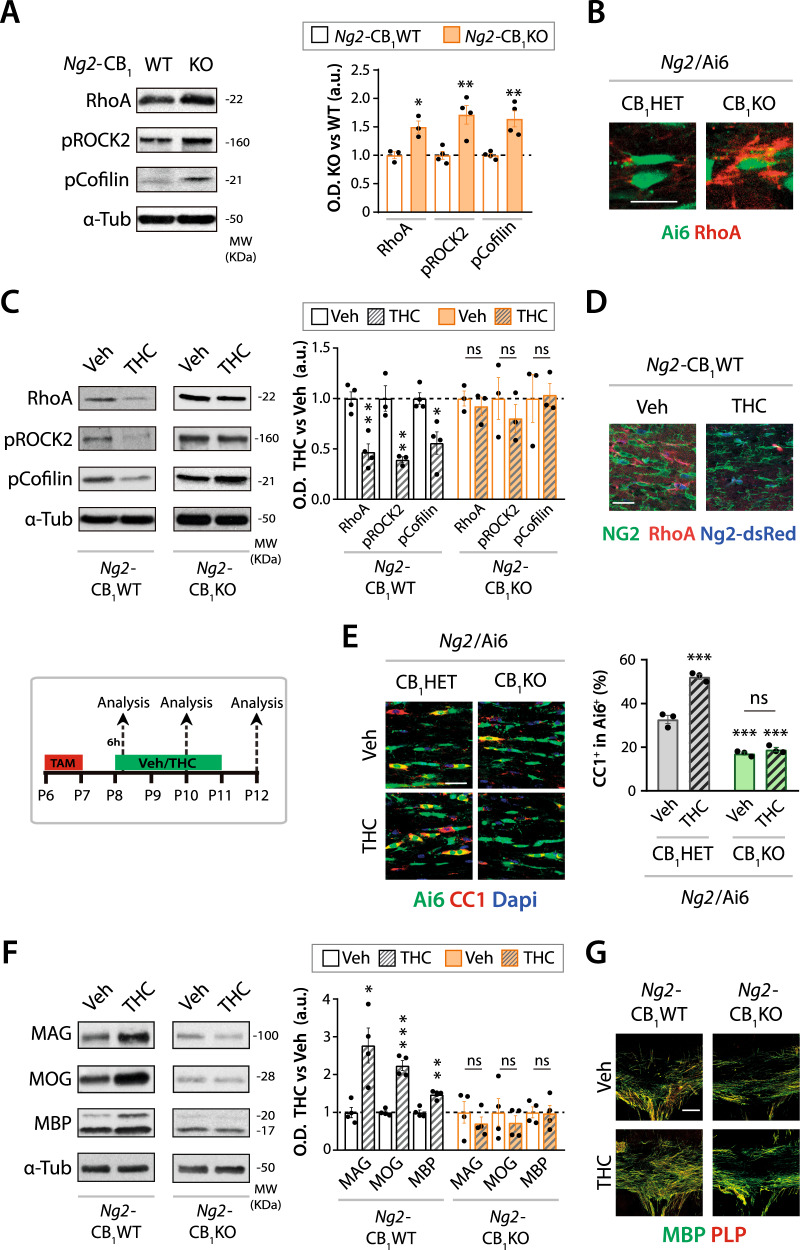
CB_1_ receptor modulates the RhoA/ROCK_2_ signaling in OPCs. Tamoxifen-driven recombination was induced at postnatal day 6 (P6-P7) in *Ng2*-CB_1_KO, *Ng2*-CB_1_WT, *Ng2*/Ai6-CB_1_KO, or *Ng2*/Ai6-CB_1_HET mice, followed by Δ^9^-Tetrahydrocannabinol (THC, 3 mg/kg) administrations the following day, for 1 time **C**, **D** or for 2 **E**, or 4 **F**, **G** consecutive days. **A** Western blot analysis for Ras homolog gene family member A (RhoA), phosphorylated RhoA/Rho-associated protein kinase 2 (pROCK_2_), and pCofilin of dissected *corpus callosum* (CC) extracts from *Ng2*-CB_1_KO and *Ng2*-CB_1_WT at P15. Quantification of optical density (O.D.) **B** Immunofluorescence analysis of RhoA expression in the recombinant Ai6^+^ population in *Ng2*/Ai6-CB_1_KO, or *Ng2*/Ai6-CB_1_HET mice at P15. **C** Western blot analysis of RhoA, pROCK2 and pCofilin protein levels of dissected CC extracts from THC or Veh-treated *Ng2*-CB_1_KO or *Ng2*-CB_1_WT mice, at 6 hours after THC administration. **D** Immunofluorescence analysis of RhoA in *Ng2*-dsRed^+^ OPCs at 6 h after THC or Veh administration. **E**-**G** *Ng2*-CB_1_KO, *Ng2*-CB_1_WT, *Ng2*/Ai6-CB_1_KO, or *Ng2*/Ai6-CB_1_HET mice were administered with THC or Veh for 2 **E** or 4 **F**, **G** days. **E** Immunofluorescence analysis and quantification of the percentage of CC1^+^ cells among the recombinant Ai6^+^ population. Western blot (**F**) or immunofluorescence (**G**) analysis of myelin-related proteins such as myelin-associated glycoprotein (MAG), myelin oligodendrocyte glycoprotein (MOG), and myelin basic protein (MBP) or proteolipid protein (PLP). Data are shown as mean ± SEM. *n* = 3 for **E**, *n* = 3–4 for **A**, **C**, *n* = 4 for **F**, independent data points were used for each experimental group. *p ≤ 0.05, **p ≤ 0.01 and ***p ≤ 0.001 vs *Ng2*-CB_1_WT or vehicle-treated groups, by two-tailed unpaired Student’s *t*-test for **A**, two-tailed unpaired Student’s or Mann Whitney test for **C** and **F**, and two-way ANOVA followed by Turkey´s multiple comparison for **E**. Scale bars, 20 µm for **B**, **D** and **E**, and 100 µm for **G**.

The Ras homolog family member A (RhoA) is a multifunctional small GTPase protein that has recently emerged as a central control point of OPC differentiation and OL maturation [22, 23]. RhoA, by stabilizing actin fibers, regulates cytoskeletal reorganization, thereby modulating the morphological changes necessary for OPC differentiation and OL maturation. Thus, activation of the RhoA/Rho-associated protein kinase (ROCK) signaling axis impedes OPC differentiation, and RhoA/ROCK inactivation promotes OPC differentiation in vitro [22, 24, 25], or under inhibitory conditions, such as in the presence of myelin [26], or following spinal cord injury [27] or hypoxia [28] in mice. Thus, we studied whether the aforementioned deficits in cell differentiation of CB_1_ receptor deficiency in OPCs may be caused by an altered RhoA signaling. Western blot analysis revealed increased RhoA protein levels and increased activation of the RhoA downstream target ROCK, paralleled by a reduced activation of cofilin, in CC extracts from *Ng2*-CB_1_KO mice (Fig. 4A). Immunofluorescence analysis in the developing CC corroborated the increased RhoA protein levels in *Ng2*/Ai6-CB_1_KO OPCs compared to their *Ng2*/Ai6-CB_1_HET controls (Fig. 4B). In line with these observations, acute THC administration (3 mg/kg, i.p.) to *Ng2*-CB_1_WT mice reduced RhoA protein and ROCK activation levels, and increased cofilin activation levels, in CC extracts (Fig. 4C). Immunofluorescence analysis in the developing CC of *Ng2*-dsRed mice confirmed the reduced RhoA protein levels in THC-treated *Ng2*-dsRed^+^ OPCs compared to Veh-treated mice (Fig. 4D).

To address the cell-autonomous action of THC in targeting RhoA selectively in OPCs through CB_1_ receptors activation to drive oligodendrogenesis, we administered THC or Veh to *Ng2*-CB_1_KO mice. THC failed to alter RhoA protein levels and activation of its downstream targets in CC extracts from *Ng2*-CB_1_KO mice compared to Veh-treated animals (Fig. 4C). Similarly, immunofluorescence (Fig. 4E, G) and western blot (Fig. 4F) analyses revealed that THC enhanced OL differentiation and increased myelin-associated protein levels in the CC of *Ng2*-CB_1_WT or *Ng2*/Ai6-CB_1_HET control mice, but not in their *Ng2*-CB_1_KO or *Ng2*/Ai6-CB_1_KO littermates. These results indicate a cell-autonomous action of THC in inducing OPC differentiation and myelination in vivo through CB_1_ receptors activation, at least in part, by targeting RhoA protein levels.

**Fig. 5:**
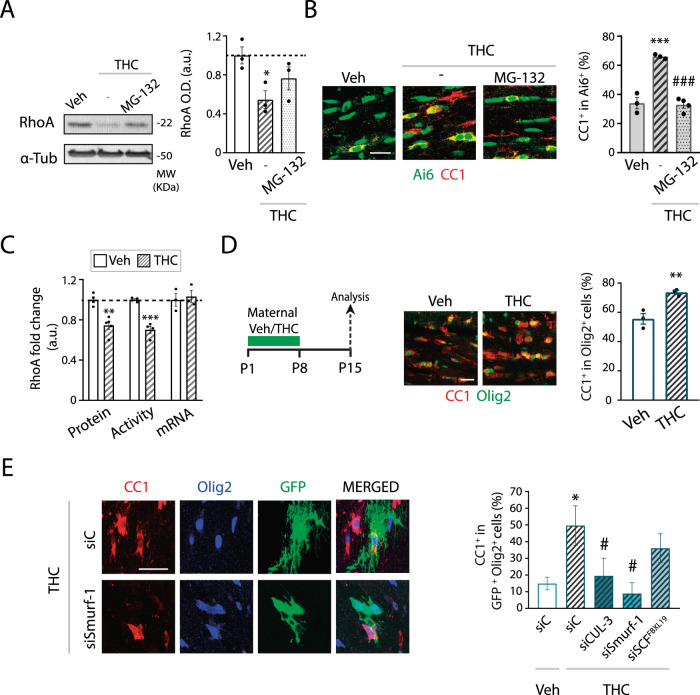
CB_1_ receptor modulates OPC differentiation through RhoA proteasomal degradation. **A**, **B** *Ng2*/Ai6-CB_1_WT mice were administered with the proteasomal inhibitor MG-132 (5 mg/kg) at 30 min before THC. **A** Western blot analysis for RhoA in dissected CC extracts at 6 hours after THC. Quantification of optical density (O.D.). **B** Immunofluorescence analysis and quantification of the percentage of Ai6^+^ cells that expressed the OL marker CC1 at 48 hours after THC administrations. **C** Relative levels of RhoA protein levels, activity, or mRNA levels in CC extracts at 6 hours after THC. **D** Maternal Tetrahydrocannabinol (THC) administration induces oligodendrocyte development in postnatal pups. THC (3 mg/kg) or Veh were administered to CD1 mouse mothers the day their pups were 1 day old, once a day for 8 consecutive days, and oligodendrogenesis was analyzed in *corpus callosum* at postnatal days 15. Immunofluorescence analysis and quantification of the percentage of CC1^+^ OLs among the Olig2^+^ population. **E** Electroporation experiments with siRNAs against Cullin-3 (CUL-3), Smurf-1 or SCF^FBXL19^, and with a GFP reporter plasmid control in Pups from Veh or THC-treated mothers. Quantification of CC1^+^ OLs among GFP^+^Olig2^+^ cells in the subcortical white matter at P30. Data are shown as mean ± SEM. *n* = 3 for **A** and **D**, *n* = 3–4 for **B**, *n* = 3–5 for **C**, and *n* = 4–9 for **E**, independent data points were used for each experimental group. *p ≤ 0.05, **p ≤ 0.01 and ***p ≤ 0.001 vs vehicle-treated groups; ^#^*p* < 0.05 and ^###^*p* < 0.001 vs THC-treated group, by one-way ANOVA followed by Tukey´s multiple comparisons for **A** and **B**, two-tailed unpaired Student’s t-test for **C** and **D**, and by one-way ANOVA Kruskal-Wallis followed by Dunn´s test for **E**. Scale bar, 20 µm for **B, D**, **E**.

Modulation of protein stability by proteasomal degradation plays an important role in RhoA biological functions [29]. To explore the possibility that CB_1_ receptors target proteasomal degradation to modulate RhoA protein levels we used a reversible proteasome inhibitor, MG-132. Thus, pretreatment with MG-132 prevented the THC-induced reduction in RhoA protein levels in CC extracts (Fig. 5A), as well as the THC-induced OL differentiation determined by immunofluorescence analysis in the CC of *Ng2*/Ai6-CB_1_WT mice (Fig. 5B). We excluded the transcriptional modulation of RhoA by CB_1_ receptors in OPCs, as we did not find differences in RhoA mRNA levels in CC extracts from acutely THC-treated mice compared to Veh-treated controls (Fig. 5C). THC-treated mice also exhibited a reduction in RhoA activity in CC extracts compared to Veh-treated controls (Fig. 5C), in line with the differences observed at RhoA protein levels. These results indicate that CB_1_ receptors modulate OPC differentiation, at least in part, by regulating RhoA proteasomal degradation.

To further study the molecular mechanism of CB_1_ receptor-mediated RhoA proteasomal degradation, we performed electroporation experiments in P2 pups from THC or Veh-treated mothers by using siRNAs against the three main E3 ubiquitin-protein ligases reported so far to target RhoA for proteasomal degradation, namely Smurf-1, Cullin-3 (CUL-3), and SCF^FBXL19^ [30–33]. First, we observed that maternal THC administration during the early postnatal period indirectly induced OPC differentiation in pups´ CC (Fig. 5D). Then, we electroporated a control GFP reporter plasmid and quantified by immunofluorescence the percentage of CC1^+^ OLs within GFP^+^Olig2^+^ cells. This analysis revealed an enhanced oligodendrogenesis in siC-electroporated pups of THC-treated mothers compared to SiC-electroporated pups from Veh-treated mothers (Fig. 5E), confirming that maternal THC administration induced OPC differentiation in control pups. Moreover, we found that Smurf-1 and CUL-3 silencing prevented the THC-induced OPC differentiation in the developing CC (Fig. 5E), indicating that CB_1_ receptor-mediated modulation of OPC differentiation requires E3 ubiquitin ligase-mediated RhoA proteasomal degradation.

### ROCK inactivation overcomes the defects in oligodendrogenesis and functional myelination of CB_1_ receptor deficient OPCs

**Fig. 6:**
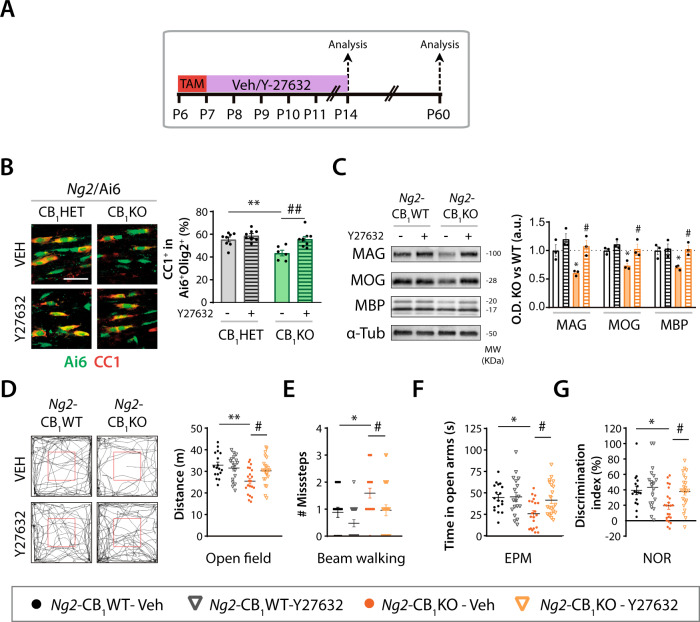
Pharmacological inactivation of ROCK overcomes the defects in oligodendrogenesis and myelination of CB_1_ deficient OPC. **A** Tamoxifen-driven recombination was induced at postnatal day 6 (P6-P7) in *Ng2*-CB_1_KO, *Ng2*-CB_1_WT, *Ng2*/Ai6-CB_1_KO and *Ng2*/Ai6-CB_1_HET mice followed by administrations of the ROCK inhibitor Y-27632 (10 mg/kg), and tissue was analyzed at P11 or P60. **B** Immunofluorescence analysis and quantification of the percentage of CC1^+^ oligodendrocytes among the recombinant Ai6^+^Olig2^+^ population at P11. **C** Western blot analysis of the myelin associated proteins, myelin-associated glycoprotein (MAG), myelin oligodendrocyte glycoprotein (MOG), and myelin basic protein (MBP) in *corpus callosum* extracts at P60. Quantification of optical density (O.D.). **D**–**G** Behavioral analysis of *Ng2*-CB_1_KO and *Ng2*-CB_1_WT mice at P60. **D** *Open field* test. Representative images of mouse trajectories. Quantification of total distance travelled. **E** *Beam walking* test. Quantification of missteps. **F** *Elevated plus maze* (EPM) test. Quantification of the time spent in open arms. **G** *Novel object recognition* (NOR) test. Quantification of the discrimination index. Data are shown as mean ± SEM. *n* = 6–9 for **B**, *n* = 3 for **C**, 16–24 for **D**–**G**, independent data points were used for each experimental group. *p ≤ 0.05, **p ≤ 0.01 vs Veh-treated *Ng2*/Ai6-CB_1_HET or *Ng2*-CB_1_WT mice; ^#^*p* ≤ 0.05, ^##^*p* ≤ 0.01 vs Veh-treated *Ng2*/Ai6-CB_1_KO mice or *Ng2*-CB_1_KO mice by two-way ANOVA followed by Tukey´s multiple comparisons. Scale bar, 20 µm in **B**.

To confirm the involvement of the RhoA/ROCK axis in the CB_1_ receptor-mediated modulation of OPC differentiation, we analyzed the effect of inhibiting ROCK pharmacologically in vivo by administering the selective inhibitor Y-27632. CB_1_ receptor deficiency was induced in *Ng2*-CB_1_KO and *Ng2*/Ai6-CB_1_KO and control mice, followed by Y-27632 or Veh administration (Fig. 6A). Immunofluorescence analysis in the developing CC confirmed a reduced proportion of CC1^+^ OLs within the Ai6^+^ population in Veh-treated *Ng2*/Ai6-CB_1_KO mice compared to Veh-treated *Ng2*/Ai6-CB_1_HET animals (Fig. 6B). Importantly, ROCK inhibition induced OPC differentiation in *Ng2*/Ai6-CB_1_KO mice, rescuing CB_1_ receptor-null OPC deficiency up to the levels found in Veh or Y-27632-treated *Ng2*/Ai6-CB_1_HET littermates (Fig. 6B). Immunofluorescence (Fig. S5) and western blot (Fig. 6C) analysis of myelin-associated proteins evidenced that ROCK blockade also restored *Ng2*-CB_1_KO myelin protein levels up to the levels of *Ng2*-CB_1_WT-Veh or Y-27632-treated mice at both, postnatal and adult ages. Notably, we also found that ROCK pharmacological blockade overcame the motor and cognitive deficits observed in *Ng2*-CB_1_KO mice at adult ages in the *open field* (Fig. 6D), *beam walking* (Fig. 6E), *elevated plus-maze* (EPM, Fig. 6F) and *novel object recognition* (NOR, Fig. 6G) tests. Altogether, these findings show that ROCK pharmacological inactivation overcomes the defects in oligodendrogenesis and functional CNS myelination of CB_1_ receptor deficient OPCs during postnatal development in mice.

## Discussion

### CB_1_ receptors modulate OPC differentiation cell-autonomously

Here, by using new conditional mouse models, we provide the first evidence for a cell-autonomous role of CB_1_ receptors in modulating OPC differentiation and OL development during postnatal myelination in mice. Our observations support the relevance of eCB signaling in the control of postnatal myelination through the activation of CB_1_ receptors located on OPCs. However, mouse OPCs express CB_1_ and CB_2_ receptors at developmental stages [9], respond to THC administration in vivo (present study), and pharmacologically blocking either of the two receptors prevents THC effects [9]. Moreover, we show that THC administration to *Ng2*-CB_1_KO mice fails to modulate oligodendrogenesis and postnatal myelination in the CC, addressing that CB_1_ receptor expression in OPCs is strictly required for cannabinoid modulation of developmental oligodendrogenesis. Thus, the involvement of CB_2_ receptors may be substantiated by the existence of a functional interaction between both receptors in OPCs, such as the CB_1_-CB_2_ heteromers observed in neuronal cells [34], or the non-cell autonomous modulation of oligodendrogenesis by CB_2_ receptors. Therefore, our study also provides evidence for a cell-autonomous action of THC administration in modulating OPC differentiation and OL development in the CC during postnatal myelination in mice, and suggests that previous pharmacological studies based on the use of synthetic cannabinoids [8], phytocannabinoids [14] or eCBs [10] may have also involved the targeting of OPCs cell-autonomously to enhance OL development during postnatal myelination.

Our results show that genetic inactivation of CB_1_ receptors in OPCs not only blocks cell differentiation, but also affects OL maturation and CNS myelination in the CC. These data are in line with previous findings in which administration of cannabinoid agonists induce OPC differentiation and enhance OL maturation and CNS myelination [8, 10, 15]. As premature and mature OLs derived from CB_1_ receptor deficient OPCs shall inherit genetic depletion of CB_1_ receptor, we cannot exclude the possibility that some of the observed maturation defects arise from an altered OPC differentiation. Thus, assessing the selective action of CB_1_ receptors in modulating OL maturation and myelin formation would require the generation of selective premature/mature OL Cre-dependent mouse models.

### CB_1_ receptor-mediated RhoA proteasomal degradation in OPCs

The CB_1_ receptor is one of the most abundant G protein-coupled receptors (GPCRs) in the mammalian brain. To date, other GPCRs, such as GPR17 [35], GPR37 [36], GPR56 [37], and GPR126 [38] have been shown to modulate OL development and/or CNS myelination. Thus, our present study adds evidence for GPCRs in modulating oligodendrogenesis and myelination. Mechanistically, GPR37 regulates OPC differentiation and CNS myelination via cAMP-dependent Ras-ERK1/2 activation [39], while GPR56 modulates OPC proliferation via Gα12/13-RhoA, without affecting OL differentiation. Here, we observed that the CB_1_ receptor-mediated modulation of OPC differentiation occurs by inducing Smurf-1/CUL-3-mediated RhoA proteasomal degradation. Thus, CB_1_ receptors may modulate RhoA proteasomal degradation by targeting both, the inactive (GDP-bound) and the active (GTP-bound) forms of RhoA by CUL_3_ and Smurf-1 respectively [29]. Indeed, pharmacological activation of CB_1_ receptor has been shown to induce RhoA proteasomal degradation in migrating pyramidal neurons during mouse corticogenesis [40]. Therefore, it is plausible to speculate that CB_1_ receptor-mediated RhoA degradation under those settings may involve Smurf-1 or CUL-3 actions (Fig. S6). Moreover, CB_1_ receptors may exert opposing effects regarding RhoA activation. CB_1_ receptors activation promote bone marrow-derived macrophage migration or phagocytosis [41–43] by increasing RhoA activity and subsequent ROCK activation via Gαi/o, but also via Gα12/13, in neurons [44] or platelets [45]. Conversely, CB_1_ receptors also inhibit carcinoma cell migration [46] by reducing RhoA activity, indicating that the differential CB_1_ receptor-modulation of RhoA signaling may depend on factors such as the cell type and the pathophysiological context.

### CB_1_ receptors in OPCs and motor and cognitive development

Here, we also reveal that CB_1_ receptor deficiency in OPCs at early postnatal ages affects the number and maturation state of the OL population in the CC in young adult mice, which is also associated with hypomyelination in several CNS regions and important behavioral deficits. In line with these results, increasing evidence has linked deregulated developmental myelination to impaired functional performance or neuropsychiatric alterations in adult mice [3, 47], such as those related with motor function [48, 49], memory [50], and anxiety-like behaviors [51–53]. Thus, it is plausible that the altered behavioral traits found in *Ng2*-CB_1_KO mice is due to a reduced number of myelinating OLs, and, thereby, to reduced myelin levels in CNS regions responsible for motor, memory, anxiety-like behaviors, which, in turn, would impact neuronal and whole-body functionality. In fact, alterations of the eCNB system during embryonic, postnatal, or adolescent ages evokes multiple long-lasting behavioral alterations in mice that persist in adulthood [54, 55]. Moreover, THC exposure during embryonic or postnatal development has been linked to psychiatric disorders, such as depression and anxiety [56, 57], spontaneous behavior, or habituation [58, 59], thus suggesting that, in addition to the neuronal component, at least part of these behavioral defects are mediated by the restricted OPC CB_1_ receptor population.

Conversely, on top of the observed behavioral alterations in complete CB_1_ receptor-deficient mice [54], the development of mouse models with CB_1_ receptors selectively deleted in dorsal telencephalic glutamatergic neurons or forebrain GABAergic neurons have contributed to dissect the populations of neuronal CB_1_ receptors responsible for the modulation of motor, memory, anxiety-like behaviors [60–62]. To sum up, this is the first study that supports the necessary role for CB_1_ receptors located on OPCs in the modulation of motor and cognitive development in mice, therefore contributing to understand the complex control of motor and cognitive development exerted by the eCB system.

### The therapeutic potential of targeting OPC CB_1_ receptors in demyelinating disorders

A number of studies have pointed out important similarities in the effects of molecules that modulate OPC differentiation during postnatal myelination and during remyelination [63, 64]. Specifically, cannabinoids modulate oligodendrogenesis under demyelinating conditions, enhancing OL regeneration, and functional CNS remyelination following cuprizone-induced demyelination [14], or in the TMEV animal model of progressive MS [12, 13], thus suggesting that CB_1_ receptors also modulate OPC differentiation under demyelinating conditions. In MS, there is a failure in OPC differentiation, which limits their remyelinating potential. Although the cause of this failure is not completely known, several studies have denoted the presence of inhibitory signals in the demyelinated CNS that prevent OPC to become mature myelinating cells. Indeed, inhibition of OPC differentiation or OL maturation by myelin debris [26], or by astrocytic chondroitin sulfate proteoglycans [27, 65], are mediated by RhoA/ROCK axis modulation [23]. Moreover, elevated levels of the eCB 2-AG upon pharmacological inhibition of MAGL reduces astrocytic chondroitin sulfate proteoglycan production and enhances OL differentiation under inhibitory conditions [12]. Thus, it is plausible that, following demyelination, CB_1_ receptor-mediated modulation of RhoA signaling controls OPC differentiation and functional remyelination in a cell-autonomous manner though OPC CB_1_ receptors, but also non-cell-autonomously through astroglial CB_1_ receptors. In any event, the CB_1_ receptor-evoked control of RhoA signaling could be potentially targeted to promote functional recovery, which would open new avenues to the therapeutic manipulation of currently intractable demyelinating diseases.

## Materials and methods

### Animal procedures

Experimental designs and procedures were approved by the Complutense University Animal Research Committee and Comunidad de Madrid, in accordance with the European Commission regulations (Directive 2010/63/EU), and addressing the issues raised in ‘Implementing guidelines on reporting research using animals (ARRIVE) guidelines. All animals used were bred into C57BL/6 J background. Animals were housed, three in a cage, in temperature-controlled rooms on an artificial 12-h light/dark cycle at 22–24 °C and relative humidity of 50–60%, with water, food availability *ad libitum* and sterile cardboard tubes as a housing refinement. All mice were pathogen free. Jackson Laboratories (Bar Harbor, ME, USA) mouse lines used: *Ng2*-dsRed (Cspg4-DsRed.T1 1Akik/J Cat# RRID:IMSR_JAX:008241) [18], *Ng2*-CreERT2 [18], *Pdgfrα*-creERT2 (RRID:IMSR_JAX:018280 [21], Rosa-Stop-Ai6, RRID:IMSR_JAX:007906) [19], CB_1_^ff^ (IMSR Cat# JAX:036107, RRID:IMSR_JAX:036107 [17] and CD1 mice. Mice were bred to finally generate *Ng2*-CB_1_KO mice (containing homozygous CB_1_-floxed/floxed alleles and heterozygous *Ng2*-CreERT2 allele), *Ng2*-CB_1_WT (containing homozygous CB_1_-floxed/floxed alleles and negative for *Ng2*-CreERT2 allele), *Pdgfrα-*CB_1_KO mice (containing homozygous CB_1_-floxed/floxed alleles and heterozygous *Pdgfrα*-CreERT2 allele), *Pdgfrα-*CB_1_WT mice (containing homozygous CB_1_-floxed/floxed alleles and negative for *Pdgfrα*-CreERT2 allele), *Ng2*/Ai6-CB_1_KO mice (containing homozygous CB_1_-floxed/floxed alleles, homozygous ROSA-stop-Ai6 alleles and heterozygous *Ng2*-CreERT2 allele), *Ng2*/Ai6-CB_1_HET mice (containing heterozygous CB_1_-floxed alleles, homozygous ROSA-stop-Ai6 alleles and heterozygous *Ng2*-CreERT2 allele), and *Ng2*/Ai6-CB_1_WT mice (containing homozygous CB_1_-WT alleles, homozygous ROSA-stop-Ai6 alleles and heterozygous *Ng2*-CreERT2 allele) (in C57BL/6 N background). Of note, *Ng2*/Ai6-CB_1_HET mice were used as controls for *Ng2*/Ai6-CB_1_KO mice, by crossing CB_1_HET x CB_1_KO mice, to obtain a higher number of littermates of the same litter, as the dynamics of oligodendrogenesis in *Ng2*/Ai6-CB_1_HET mice were similar to *Ng2*/Ai6-CB_1_WT mice in immunofluorescence studies (not shown). Delta-9-tetrahydrocannabinol (THC, 3 mg/kg), MG-132 (5 mg/kg, Medchem, NJ, USA), or vehicle, were dissolved in 100 μL Tween-80/NaCl (1:18, v/v) and 1% (v/v) of dimethyl sulfoxide [9]. MG-132 was administered intraperitoneally 30 min before THC. In maternal THC administration experiments, we confirmed CB_1_ receptors activation in the CNS of pups at 4 h after administering THC or Veh to mothers by analyzing pS6 and cFos levels (not shown). Y27632 (10 mg/kg, Medchem, Monmouth Junction, NJ, USA) was dissolved in saline and administered intraperitoneally. Recombination was induced by tamoxifen (Tam; 24 h apart; 37.5 mg/kg i.p. dissolved in Ethanol-sunflower oil 1:9). Experiments included male and female mice.

### Fluorescent-activated cell sorting

Rosa-Ai6^+^ cells were isolated from Ng2/Ai6-CB1KO and Ng2/Ai6-CB1HET at postnatal day 10, after two tamoxifen injections (37.5 mg/kg), at P6 and p7, as previously described [20]. Briefly, CC was dissected following a second step of dissociation using papain (30 μg/ml in DMEM-Glutamax, with 0.24 μg/ml L-cystein and 40 μg/ml DNase I) and cells were put on a preformed 30% Percoll density gradient before centrifugation for 15 min. Rosa-Ai6^+^ cells were FACS sorted on an FACS Aria II (BD Biosciences). Propidium iodide (PI) was used to exclude dead cells. For genomic analysis, cells were washed twice in PBS 1×, then the dry cell pellets were frozen at −80 °C.

### Electroporation experiments

siRNAs purchased from Santa Cruz (Dallas, TX, USA), siC (SC-37007) siSmurf-1 (ref: sc-41674), siCUL-3 (ref: sc-35131), SCF^FBXL19^ (39393) were electroporated into postnatal day 2 (P2) CD1 pups. Vectors were diluted at 1 μg/μl in PBS with 2.5 mg/ml fast green (Sigma, St Louis, MO, USA) and injected, together with a constitutive GFP overexpression plasmid (pCAG-GFP), in the lumen of the diencephalic ventricle. Electroporation was performed with a BTX electroporator (Holliston, MA, USA) with these parameters: 5 pulses, intensity = 95 V, pulse length = 50 ms, inter-pulse interval = 50 ms. The same day of electroporation, mothers received THC (3 mg/kg) or Veh administrations, once a day for 14 days. Efficient silencing of ubiquitin ligases was confirmed by immunofluorescence analysis at 4 days after electroporation, by using anti-Smurf-1, anti-CUL-3 and anti-SCF^FBXL19^ antibodies for each experimental condition (not shown).

### Immunofluorescence

Brain tissue was processed as previously described [66]. Briefly, mice were perfused transcardially with 4% paraformaldehyde (PFA), and brains postfixed overnight in 4% PFA and treated with 30% sucrose before freezing. 30-μm-thick coronal free-floating brain cryosections were washed in PBS, blocked with 10% goat serum, and incubated with the indicated primary antibodies (overnight at 4 °C). When needed, antigen retrieval immunostaining was performed with citric acid (10 mM, pH 6, 65 °C for 30 min) or with retrieve-all antigen unmasking system 3. Acidic (Biolegend) at 92 °C for 10 min. For immunofluorescence analysis in vitro, CC extracts from P10 *Ng2*/Ai6-CB_1_KO and *Ng2*/Ai6-CB_1_WT mice administered with tamoxifen (37.5 mg/kg) at P6 and P7, was dissociated, and individual cells were attached to glass slides by cytospin. After air-drying, slides were treated with 4% PFA for 10 minutes. The primary antibodies used are as follows, anti-CB1R (1:500, Frontier Institute, CB_1_-GP-Af530), anti-CC1 (1:400, Millipore, Cat#OP80, RRID:AB_2057371), anti‐Olig2 (1:250, Millipore Burlington, MA, USA) Cat# AB9610, RRID:AB_570666), anti‐GSTπ (1:200, MBL International Cat# 312, RRID:AB_591792, Wobrun, MA, USA), anti-MBP (1:200, BioLegend, (San Diego, CA, USA) Cat# 836504, RRID:AB_2616694), anti-NG2, (1:200, MBL International, Cat# AB5320, RRID:AB_11213678), anti‐PLP (1:500, Abcam (Waltham, MA, USA), Cat#ab28486, RRID:AB_776593), RhoA (1:500, Cytosqueleton (Denver, CO, USA) Cat# ARH03, RRID:AB_10708069), Nav1.6 (1:300, Alobome labs (Jerusalem, Israel), Cat# ASC-009, RRID:AB_2040202), Caspr (1:300, Millipore, Cat# MABN69, RRID:AB_10806491) FluoroMyelin (1:300, Thermo Fisher Scientific (Waltham, MA, USA) Cat# F34652, RRID:AB_2572213). Nuclei were visualized with DAPI. The appropriate mouse, rabbit or guinea pig highly cross-adsorbed Alexa Fluor 488, 594, and 647 secondary antibodies (1:1000, Invitrogen, Waltham, MA, USA) were used.

### Confocal microscopy

Optical sections (z = 0.5 µm) of confocal epifluorescence images were sequentially acquired using a confocal laser-scanning microscope TCS-SP8 (20×, 40× and 63×, Leica DMI6000 B instrument) and Leica Application Suite X (LAS X) software. Images were acquired in the CC of each animal, a minimum of six correlative slices from a 1-in-10 series located between +0.7 and −0.5 mm from bregma were analysed. Cell counts were performed blindly using ImageJ software (NIH) in the *corpus callosum* (CC) and data are presented as the mean cell number per mm^2^ or as a percentage of positive cells. For CB_1_ inmunostaining of cytospined cells, the microscope was configured to capture 16 images following the z axis, which resulted in 8 µm thick stacks.

### Electron microscopy

Mice were transcardially perfused with PBS followed by a fixative solution containing 4% PFA, 2.5% glutaraldehyde, and 0.5% NaCl in 0.1 M PB, as described [67]. Brains were postfixed overnight at 4 °C and stored in 1% paraformaldehyde. Vibratome sections (100 µm) containing the CC were cut in the coronal plane on a vibratome (VT1000S, Leica (Wetzlar, Germany) and incubated in 1% OsO4, then embedded in epoxy resin overnight to polymerize at 60˚C, and then trimmed and glued onto epoxy resin capsules. Semi‐thin sections (500 nm) were cut using a Power Tome ultramicrotome (RMC Boeckeler) and stained with 1% toluidine blue. Ultrathin (60 nm) sections were cut with a diamond knife (Diatome), collected on nickel mesh grids, and stained with 4% uranyl acetate and 2.5% lead citrate for electron microscope visualization. Electron microscopy images of the rostral CC were taken from randomly selected fields with a Jeol JEM Plus electron microscope at the Service of Analytical and High-Resolution Microscopy in Biomedicine of University of the Basque Country UPV/EHU. The mean number of myelinated axons was analyzed in 10 non-serial electron micrographs per animal taken systematically at a magnification of 5,000×.

### Sudan black

Floating sections were mounted on to *TESPA*-coated *glass slides*, dehydrated in 70% ethanol and stained with 0.5% Sudan Black in 70% ethanol for 20 min. Excess staining was removed by washing the slides in 70% ethanol and finally rinsed with water. Samples were observed under light microscopy in a Zeiss Axioplan2 microscope.

### Western blot

CC tissue was microdissected from 500-μm-thick coronal sections and proteins were extracted using RIPA buffer (SDS 0.1%, Sodium deoxycholate 0.5%, NP40 1%, NaCl 150 mM, Tris-HCl 50 mM pH8 in PBS) containing PMSF, protease inhibitors, and sodium orthovanadate. Protein samples were separated on 12% acrylamide (Bio-Rad) gels and transferred to polyvinylidene difluoride (PVDF membranes (Millipore). Membranes were placed in blocking buffer (5% w/v BSA in TBS-T + Azida 0.02%) and probed with primary antibodies overnight at 4 °C. The primary antibodies used are as follows: anti-MAG (1:1000, Abcam, Cat#ab89780, RRID:AB_2042411), anti-MOG (1:2000, Abcam Cat#ab32760, RRID:AB_2145529), anti-MBP (1:1000, BioLegend, Cat#836504, RRID:AB_2616694), RhoA (1:500, Cytosqueleton Cat# ARH03, RRID:AB_10708069), pROCK_2_ (1:750, Genetex, Hsinchu City, Taiwan Cat# GTX122651, RRID:AB_2560946), pCofilin (1:500, Cell Signaling Technology Cat# 3313, RRID:AB_2080597) and anti-α-tubulin (1:5000, Sigma-Aldrich, Cat#T9026, RRID:AB_477593). After incubation with corresponding HRP-conjugated secondary antibody proteins were visualized using an enhanced chemiluminescence substrate mixture (ECL Plus; GE Healthcare; Santa Cruz Biotechnology; 1:5000). Band intensity of films was quantified using Adobe Photoshop software. Protein levels were normalized to the internal control α-tubulin. See full length uncropped original western blots in supplemental material.

### Quantitative PCR

RNA was isolated using RNeasy Plus kit (Quiagen). cDNA was obtained with Transcriptor (Roche). Real-time quantitative PCR (qPCR) assays were performed using the FastStart Master Mix with Rox (Roche, Basel, Switzerland) and probes were obtained from the Universal Probe Library Set (Roche). Amplifications were run in a 7900 HT-Fast Real-Time PCR System (Applied Biosystems). The sequence of primers used are as follows: F-β-ACTIN: AAGGCCAACCGTGAAAAGAT; R-β-ACTIN: GTGGTACGACCAGAGGCATAC; F-MAG: GGTGTTGAGGGAGGCAGTTG; R-MAG: CGTTGTCTGCTAGGCAAGCA; F-MBP: GGAAGGCAGGTGATGGTTGA; R-MBP: ACACTGGAGGGCAAACACTC; F-MOG: TCCATCGGACTTTTGATCCTCA; R-MOG: GCTCCAGGAAGACACAACCA; F-RhoA: GAATGACGAGCACACGAGAC; R-RhoA: AAAAGCGCCAATCCTGTTT; F-TBP: GGGGAGCTGTGATGTGAAGT; R-TBP: CCAGGAAATAATTCTGGCTCA Each value was adjusted by using β-actin and TBP mRNA levels as reference.

### Genomic recombination analysis

Recombination was tested by PCR with genomic DNA isolated from sorted Rosa-Ai6 cells by the use of the GenElute mammalian genomic DNA Miniprep Kit (Sigma-Aldrich), as previously described [68]. PCR was performed using Taq DNA polymerase (Thermo Fisher Scientific, Inc.) For the recombined CB1 allele we used the forward primer 5′-GCTGTCTCTGGTCCTCTTAAA-3′ (G50) and the reverse primer 5′-CTCCTGTATGCCATAGCTCTT-3′ (G53) resulting in a 600 bp fragment.

### RhoA activity essay

Dissected *corpus callosum* extracts were processed for active RhoA quantification with the G-LISA kit (Cytoskeleton, Cat#BK124) following manufacturer’s guide.

### Behavioral assessments

All tests were conducted during the light cycle, with uniform lighting conditions and white noise in an isolated room. Animals were acclimated to the room for 45–60 min before testing. *Beam walking test:* To evaluate fine motor coordination, mice were trained to cross a narrow wood beam (100 cm length, 10 and 7 mm width) [69]. *Open-field test:* Mice were placed in the center of an open-field arena (70 × 70 × 40 cm) and allowed to freely explore it for 5 min. Behavior was recorded with a video camera placed above and the video tracking software SMART 3.0 (Panlab, Spain) was used for analysis [70]. *Novel Object Recognition test* (NOR): The day before the test, mice were habituated to an empty open-field arena for 10 min. The day of the test, mice were allowed to freely explore the arena with two identical objects for 10 min and, after 2 hours, mice could explore the arena containing a familiar object and a novel object. Discrimination index was calculated as the difference in exploration time between the novel and familiar object divided from the total exploration time and expressed as a percentage. Preference Index was calculated as the percentage of exploration time spent examining the novel object over the total exploration time. *Modified Y-Maze test*: Mice were allowed to explore the maze with one of the three arms closed for 3 min. After an inter-trial interval of 5 min, mice could freely explore all three arms of the maze for 3 min, and the time spent in each arm was registered. *Actimeter/ Locomotor activity test:* Spontaneous motor activity was evaluated using an automated actimeter (Acti-Track; Panlab, Barcelona, Spain). This consisted of a 22.5 × 22.5 cm area with 16 surrounding infrared beams coupled to a computerized control unit. After 1 min of habituation, activity was recorded for a period of 5 min and data were collected with Acti-Track v2.7 software (Panlab, Barcelona, Spain). *Elevated plus-maze test* (EPM): Mice were placed in the center of the maze (two open arms and two closed arms of 30 ×7 cm arranged orthogonally 60 cm above the floor) and allowed to explore it for 5 min. The test was conducted under red light conditions.

### Statistics

The n number of animals per group or experiments per condition is indicated in every case. The declared group size is the number of independent data points, and that statistical analysis was done using these independent data points. Studies were designed to generate groups of equal size, using randomisation and blinded analysis. The numbers illustrated represent the animals used in each of the experiments, after considering any unexpected loss of data or exclusion criterion. In some cases, experimental losses may be determined by animals receiving the wrong treatment, infections unrelated to the experiment, sampling errors (e.g., inadequate calibration of equipment, software error during acquisition), or other human error (e.g., forgetting to switch on equipment). Power analysis was conducted with IBM SPSS software (IBM France, Bois-Colombes, France). Sample sizes were based on our prior studies where similar sample sizes were adequately powered to detect significant differences. All variables were first tested for both, normality (Shapiro test or D’Agostino & Pearson normality test with *p* > 0.05) and homogeneity of variances (Brown-Forsythe test with *p* > 0.05). When comparing two groups we use unpaired two-tailed t-test for normal distribution (unpaired *t*-tests were Welch-corrected if needed) or Mann-Whitney test when they did not distribute normally. For comparisons of more than two groups, if data were found to be normally distributed, one-way ANOVA followed by Tukey’s post hoc test was carried out. The post hoc tests were conducted only if F in ANOVA achieved *P* < 0.05 and there was no significant variance in homogeneity. If data were found to not be normally distributed, then a Kruskal–Wallis one-way ANOVA with uncorrected Dunn’s post hoc test was carried out. For data from Figs. 4E and 6, two-way ANOVA followed by Tukey’s post hoc test was carried out. Differences with *p* < 0.05 between group means were considered statistically significant. All data analyses were done using GraphPad Prism 7.00.

## Supplementary information

Supplementary information- Supplementary figures

Supplemental information original western blot scans

Data sets

Check list

## Data Availability

All data generated or analyzed during this study are available in the supporting information.
