# Supplementary material for: Cannabinoid CB1 receptor gene inactivation in oligodendrocyte precursors disrupts oligodendrogenesis and myelination in mice
Source: Cell Death Dis. 2022 Jul 7;13(7):585. doi: 10.1038/s41419-022-05032-z (PMC9263142; doi:10.1038/s41419-022-05032-z)
Supplement: Supplementary file 1 — Supplementary information- Supplementary figures [file 41419_2022_5032_MOESM1_ESM.pdf]

**Supplemental Information for**

# Cannabinoid $CB_1$ receptor gene inactivation in oligodendrocyte precursors disrupts oligodendrogenesis and myelination in mice

Aníbal Sánchez-de la Torre, Tania Aguado, Alba Huerga-Gómez, Silvia Santamaría, Antonietta Gentile, Juan Carlos Chara, Carlos Matute, Krisztina Monory, Susana Mato, Manuel Guzmán, Beat Lutz, Ismael Galve-Roperh & Javier Palazuelos

Corresponding author: Javier Palazuelos

Email: j.palazuelos@ucm.es

**This PDF file includes:**

Supplementary Figures S1 to S6

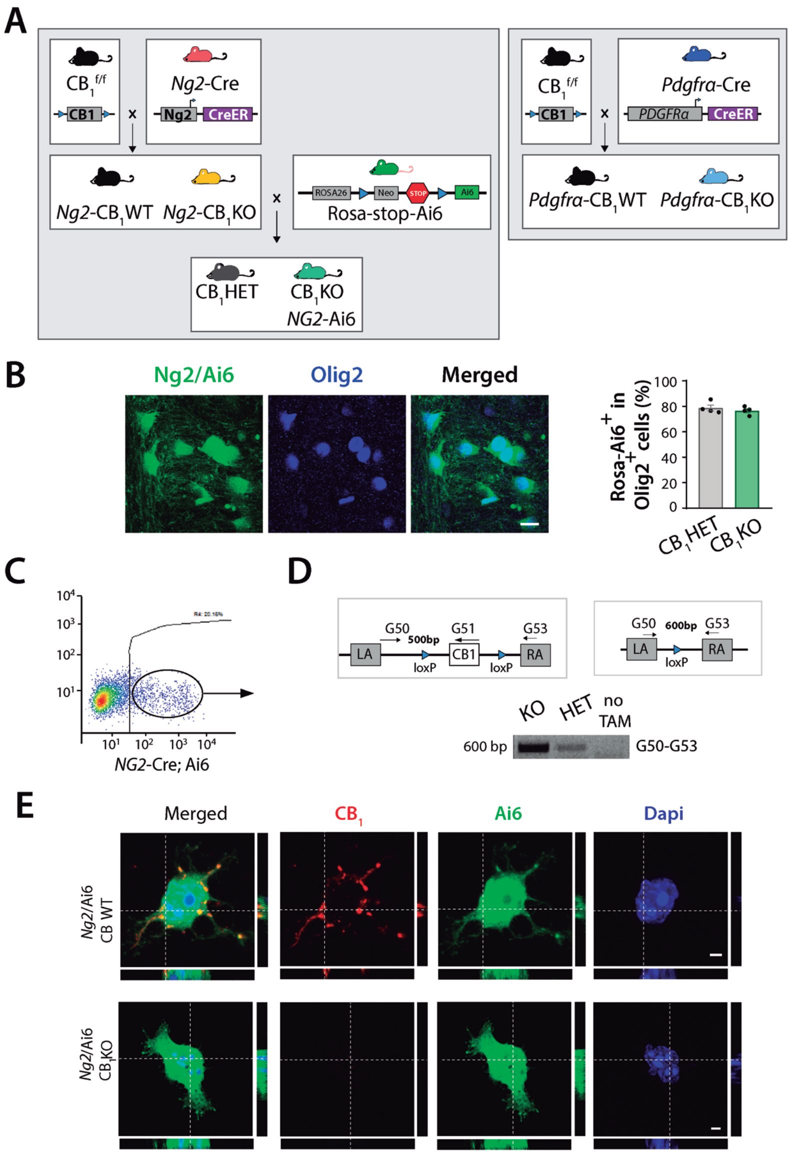


Fig. S1

**Fig. S1. Selective $CB_1$ receptor gene inactivation in oligodendrocyte precursor cells *in vivo*.** **A** Diagram of the generation of the *Ng2*/Ai6-$CB_1$KO and *Pdgfrα*-$CB_1$KO mouse lines. **B** Recombination efficiency was determined by immunofluorescence analysis in the *corpus callosum* at postnatal day 11, following tamoxifen-driven recombination at postnatal day 6 (P6, P7). Representative images and quantification of the percentage of Rosa-$Ai6^+$ cells among $Olig2^+$ cells. **C** Representative image of FAC-sorting analysis of *Ng2*/Ai6-$CB_1$KO and control *Ng2*/Ai6-$CB_1$HET cells. **D** Genetic recombination of the $CB_1$ locus in the $Ai6^+$ cell population was confirmed by genomic DNA extraction of FAC-sorted *Ng2*/Ai6-$CB_1$KO and control cells. **E** Immunofluorescence analysis of $CB_1$ receptors in *Ng2*/Ai6-$CB_1$WT and *Ng2*/Ai6-$CB_1$KO cells obtained from the CC and attached to glass slides. Representative confocal images showing orthogonal plane. Data are shown as mean +/- SEM. n = 4 for **B** independent data points used per experimental group. Scale bars, 20 µm.

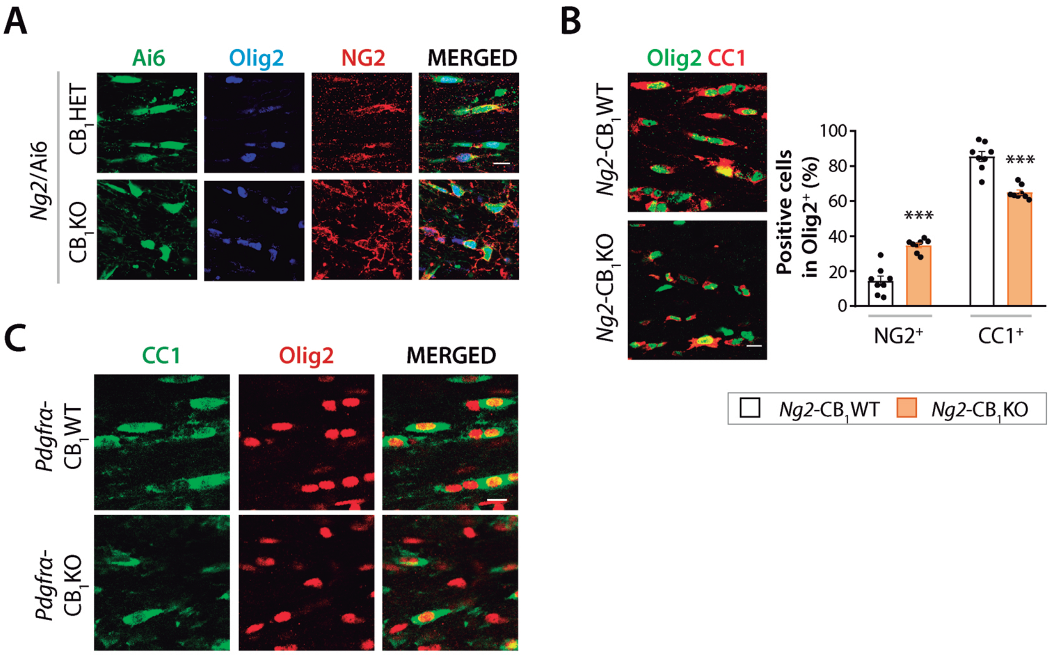


Fig. S2

**Fig. S2. $CB_1$ receptor gene inactivation in OPC blocks cell differentiation.** Tamoxifen-driven recombination was induced at postnatal day 6 (P6-P7) in *Ng2*/Ai6-$CB_1$HET and *Ng2*/Ai6-$CB_1$KO mice (**A**), *Ng2*-$CB_1$KO (**B**), or *Pdgfrα*-$CB_1$KO and control mice (**C**), and oligodendrogenesis was analyzed in the *corpus callosum* at P15 (**A** and **C**) and P60 (**B**). Representative confocal microscopy in the *corpus callosum* immunostained with the indicated antibodies (**A** and **C**). **B** Immunofluorescence analysis and quantification of the percentage of $NG2^+$ OPCs or $CC1^+$ OLs among $Olig2^+$ cells in *Ng2*-$CB_1$KO and *Ng2*-$CB_1$WT mice at P60. Data are shown as mean +/- SEM. n = 8 for **B**, independent data points used per experimental group, ***$p \leq 0.001$ vs *Ng2*-$CB_1$WT mice by two-tailed unpaired Student's t-test. Scale bars, 20 µm.

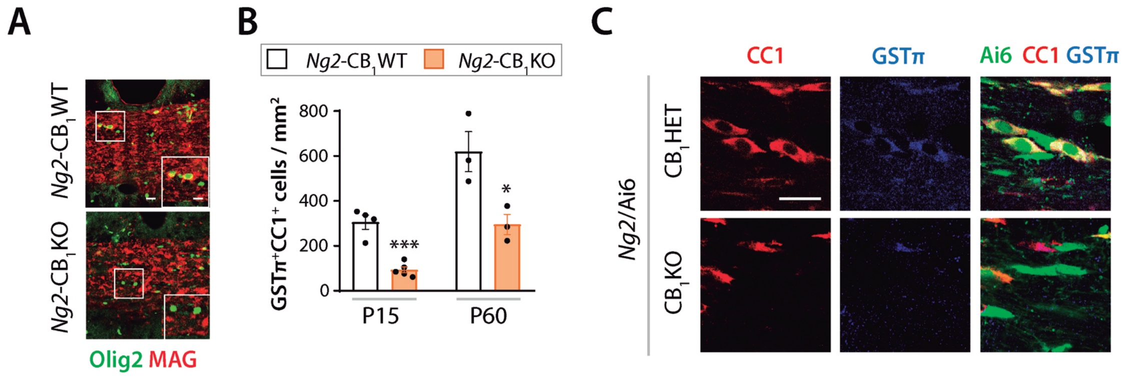


Fig. S3

**Fig. S3. $CB_1$ receptor gene inactivation in OPC disrupts oligodendrocyte maturation.** Tamoxifen-driven recombination was induced at postnatal day 6 (P6-P7) in *Ng2*-$CB_1$KO and *Ng2*-$CB_1$WT mice *(***A** and **B**), or *Ng2*/Ai6-$CB_1$KO and *Ng2*/Ai6-$CB_1$HET mice (**C**) mice, and oligodendrocyte maturation was analyzed in the *corpus callosum* (CC) at P15 (**B** and **C**) and P60 (**A** and **B**). **A** Representative confocal microscopy images in the CC immunostained with MAG and Olig2 antibodies. **B** Immunofluorescence analysis and quantification of the density of $GST\pi^{+}CC1^{+}$ mature OLs. **C** Representative confocal images of indicated antibodies. Data are shown as mean +/- SEM. n = 3-5 for **B**, independent data points used per experimental group, *$p \leq 0.05$, **$p \leq 0.01$ vs *Ng2*-$CB_1$WT mice by two-tailed unpaired Student's t-test. Scale bars, 20 µm for **A** and **C**, 10 µm for inset in **A**.

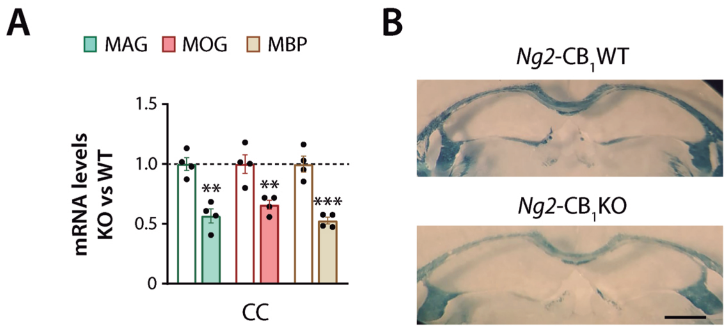


Fig. S4

**Fig. S4. $CB_1$ receptor gene inactivation in OPCs causes hypomyelination in young adult mice.** Tamoxifen-driven recombination was induced at postnatal day 6 (P6-P7) in *Ng2*-$CB_1$KO and *Ng2*-$CB_1$WT mice and myelination was analyzed at P60. **A** qPCR analysis of myelin-associated mRNA levels in the CC at P60. **B** Sudan Black staining at the caudal CC. Data are shown as mean +/- SEM. n = 4 for **A**, independent data points used per experimental group, **$p \leq 0.01$, ***$p \leq 0.001$ vs *Ng2*-$CB_1$WT mice by two-tailed unpaired Student's t-test. Scale bars 600 μm for **B**.

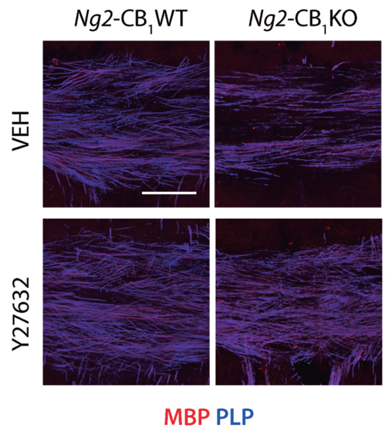


Fig. S5

**Fig. S5. Pharmacological ROCK inactivation overcomes the defects in developmental myelination of $CB_1$ deficient OPCs at postnatal ages.** Tamoxifen-driven recombination was induced at postnatal day 6 (P6-P7) in *NG2*-$CB_1$KO and *NG2*-$CB_1$WT mice followed by administrations of the ROCK inhibitor Y-27632 (10 mg/kg), and tissue was analyzed at P11. Representative images of immunofluorescence analysis for the myelin-associated proteins proteolipid protein (PLP) and myelin basic protein (MBP). Scale bar, 80 µm.

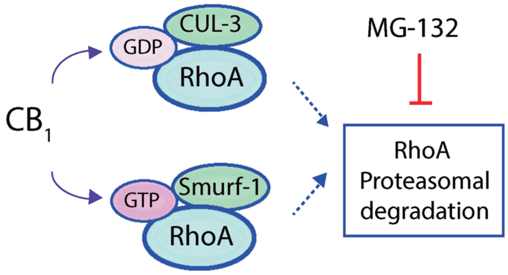


Fig. S6

**Fig. S6. Diagram representing the proposed model of CB1 receptor modulation of RhoA proteasomal degradation**. CB1 receptor, by inducing Smurf-1/CUL-3 mediated RhoA proteasomal degradation, modulates OPC differentiation.
