# Supplementary material for: Cannabinoid CB1 receptor gene inactivation in oligodendrocyte precursors disrupts oligodendrogenesis and myelination in mice
Source: Cell Death Dis. 2022 Jul 7;13(7):585. doi: 10.1038/s41419-022-05032-z (PMC9263142; doi:10.1038/s41419-022-05032-z)

Supplemental Information for

Cannabinoid CB_1 receptor gene inactivation in oligodendrocyte precursors disrupts oligodendrogenesis and myelination in mice

Aníbal Sánchez-de la Torre, Tania Aguado, Alba Huerga-Gómez, Silvia Santamaría, Antonietta Gentile, Juan Carlos Chara, Carlos Matute, Krisztina Monory, Susana Mato, Manuel Guzmán, Beat Lutz, Ismael Galve-Roperh & Javier Palazuelos

Corresponding author: Javier Palazuelos

Email: j.palazuelos@ucm.es

This PDF file includes:

Original Western blot scans

Biological replicates of western blot analysis shown in Figure 2A

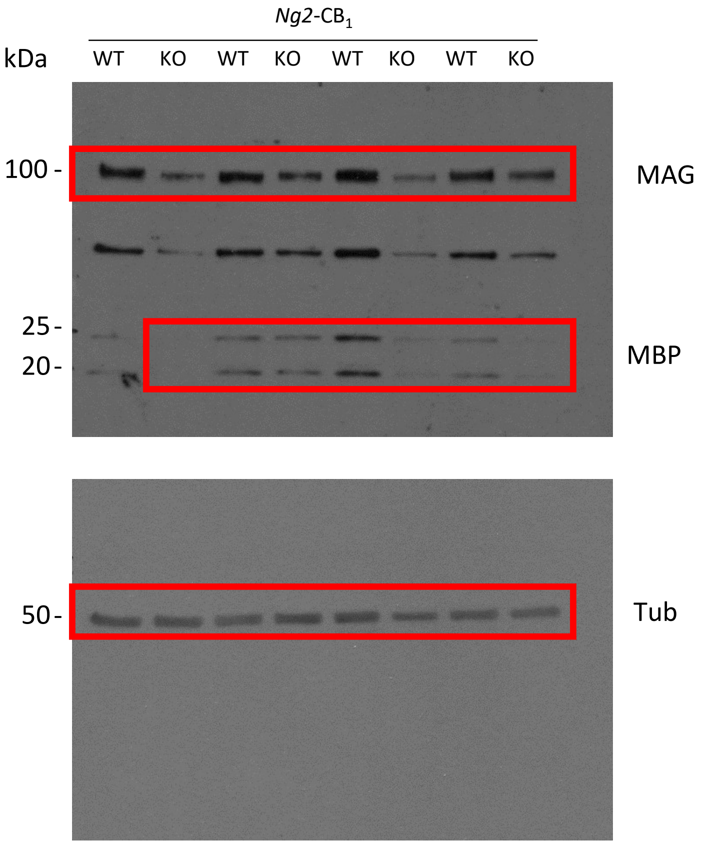

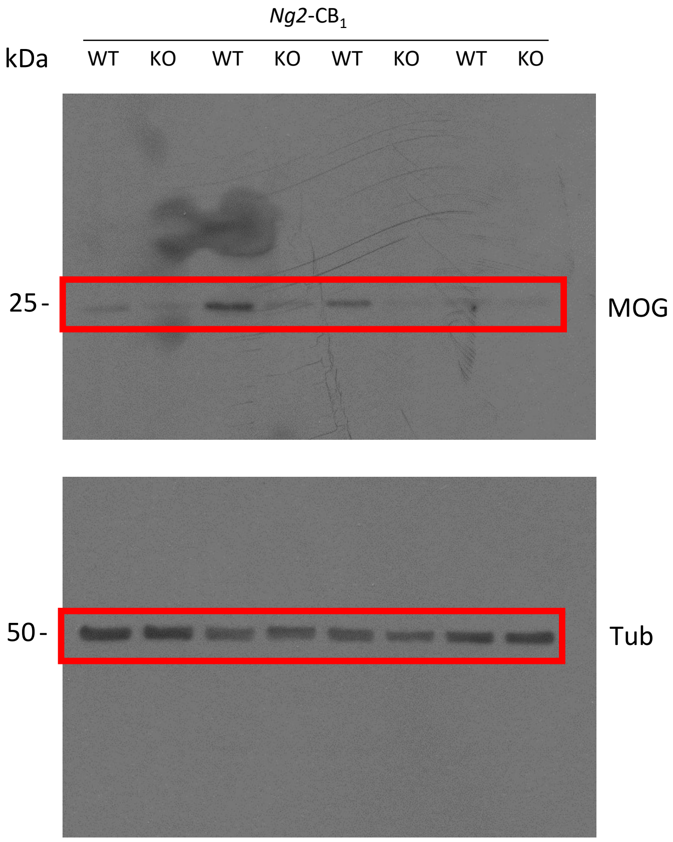

Biological replicates of western blot analysis shown in Figure 2A

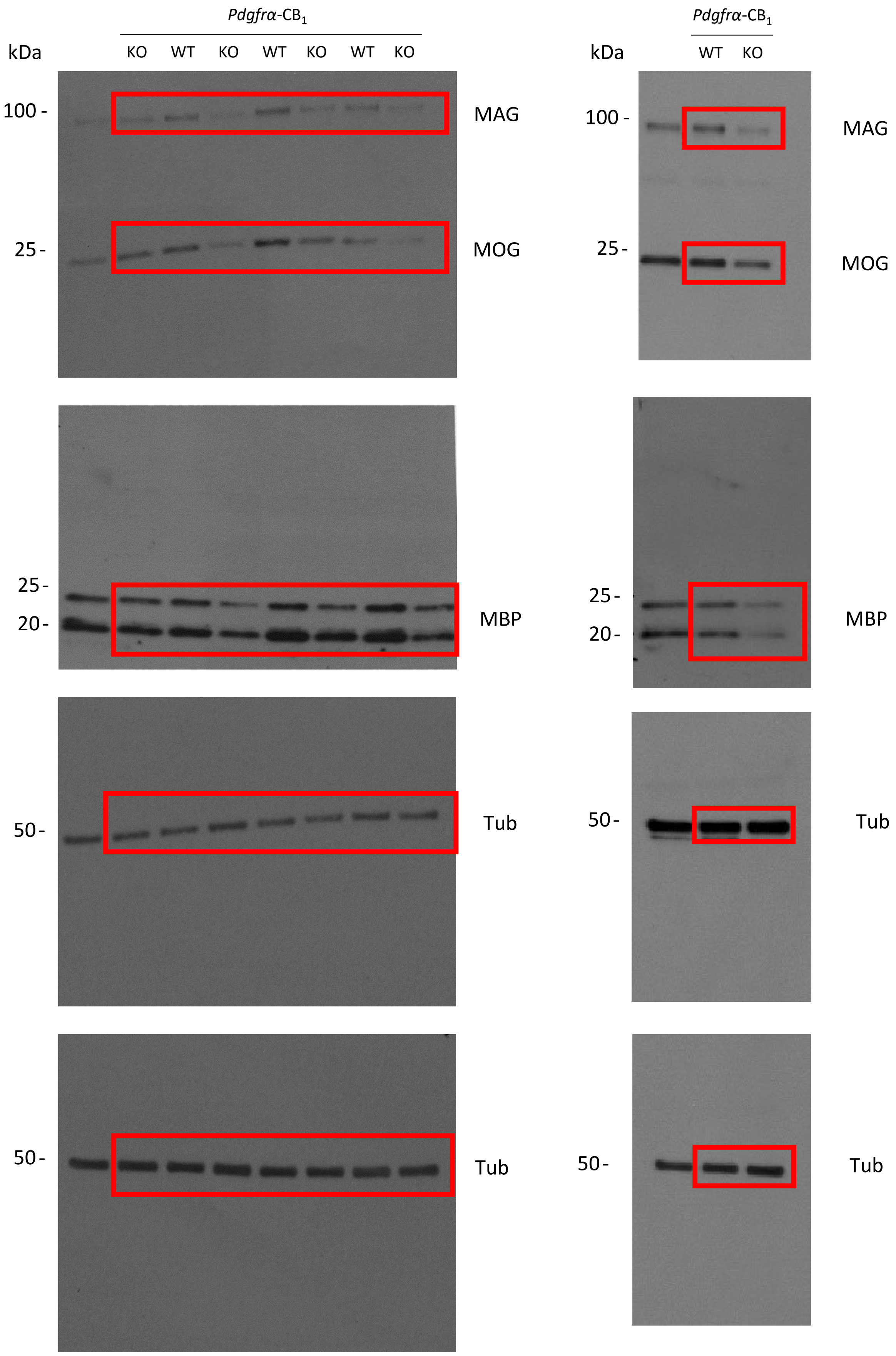

Biological replicates of western blot analysis shown in Figure 2F

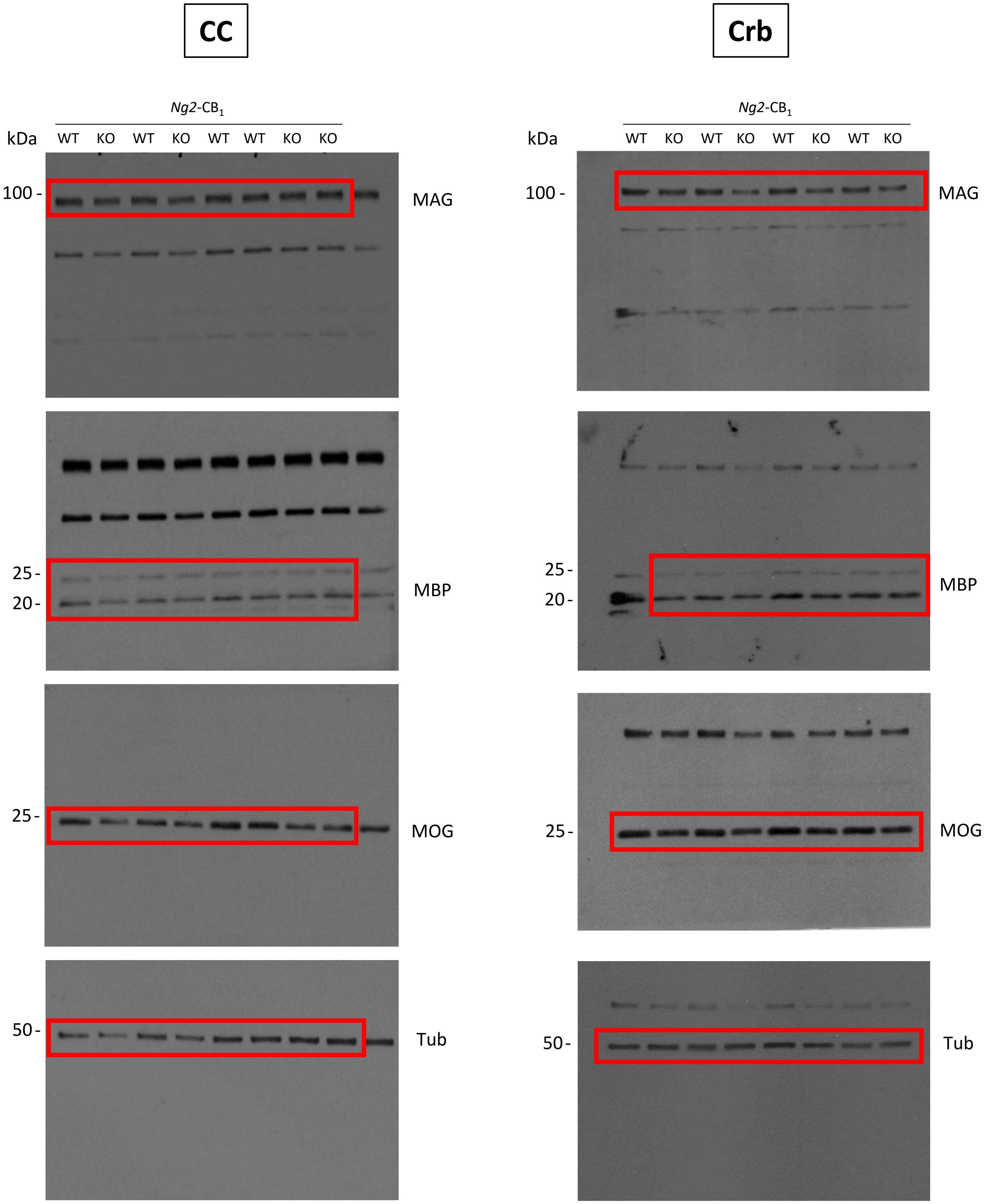

Biological replicates of western blot analysis shown in Figure 2F

Hipp

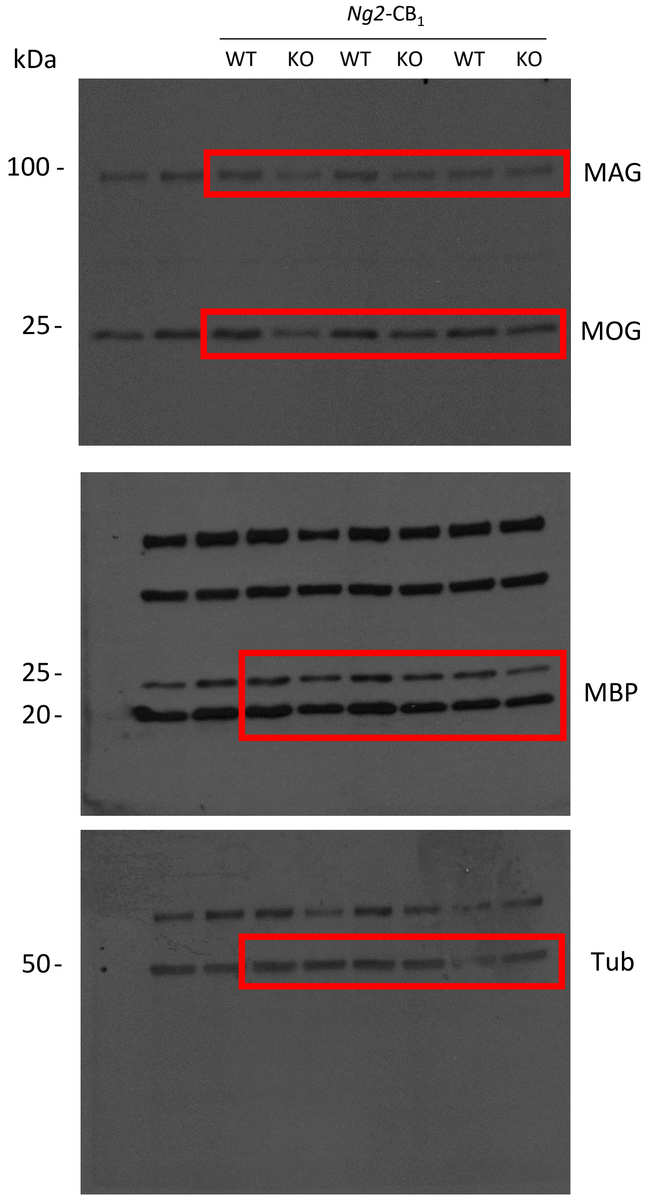

Biological replicates of western blot analysis shown in Figure 2F

mPFC

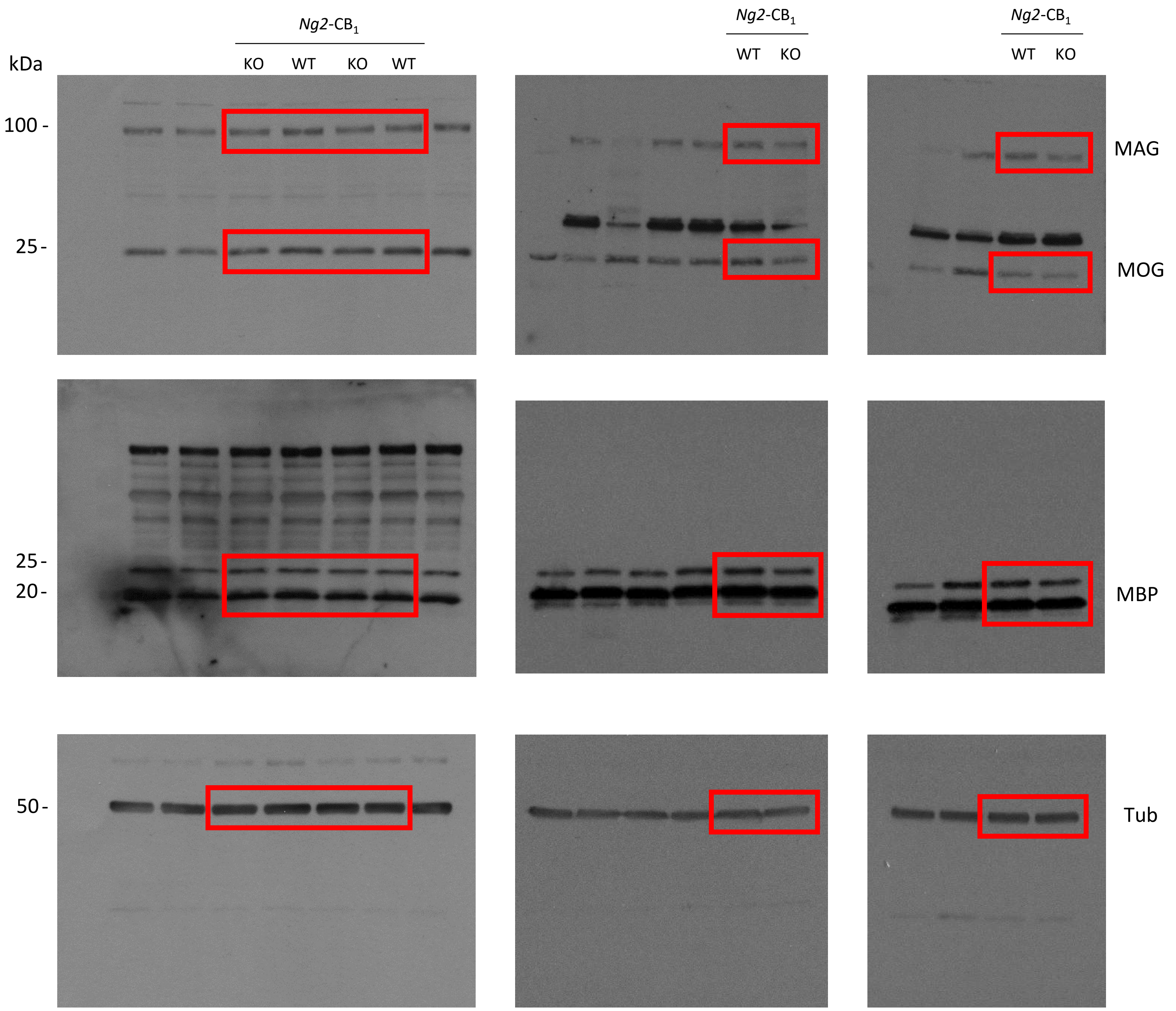

Biological replicates of western blot analysis shown in Figure 4A

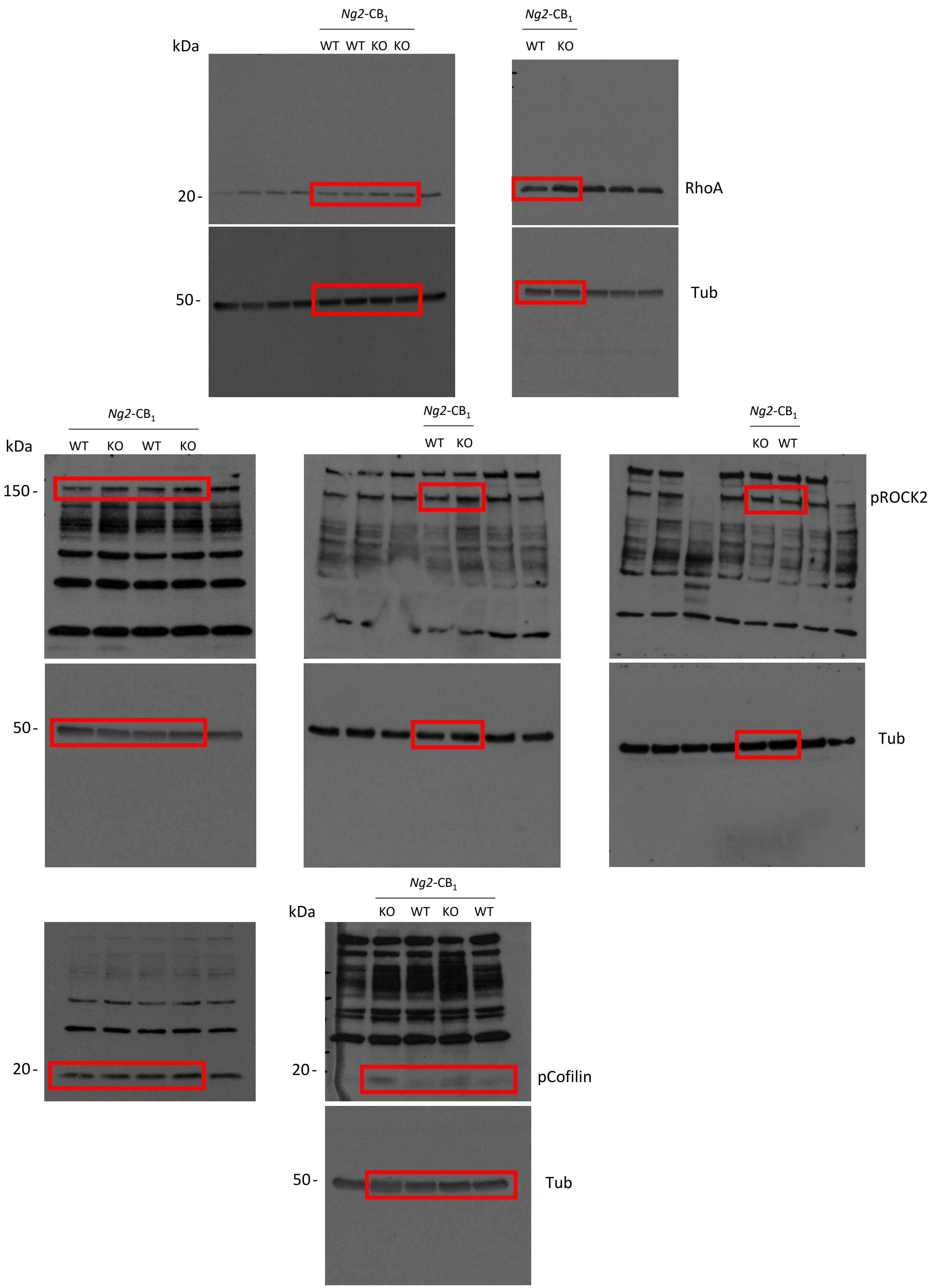

Biological replicates of western blot analysis shown in Figure 4C

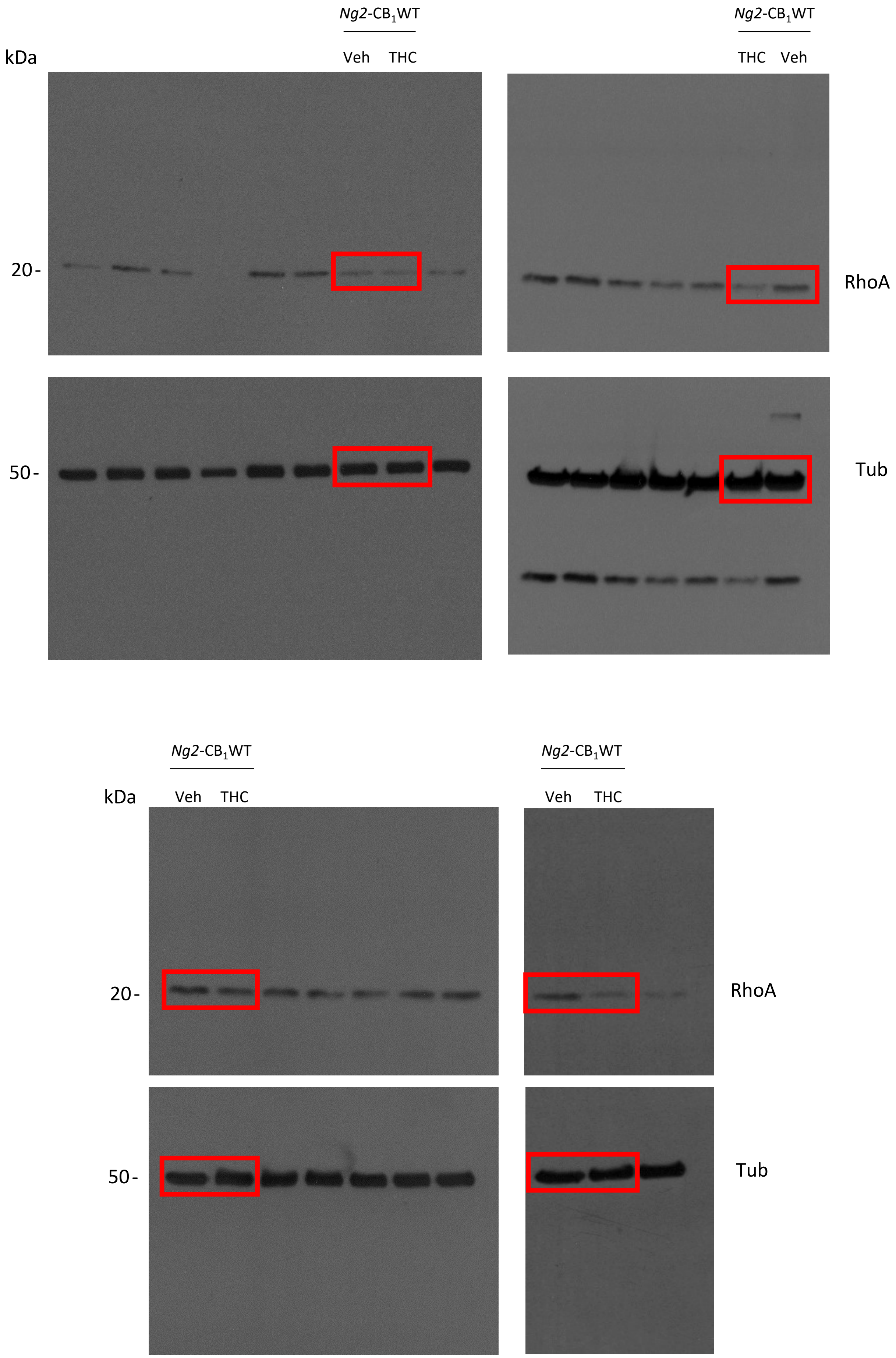

Biological replicates of western blot analysis shown in Figure 4C

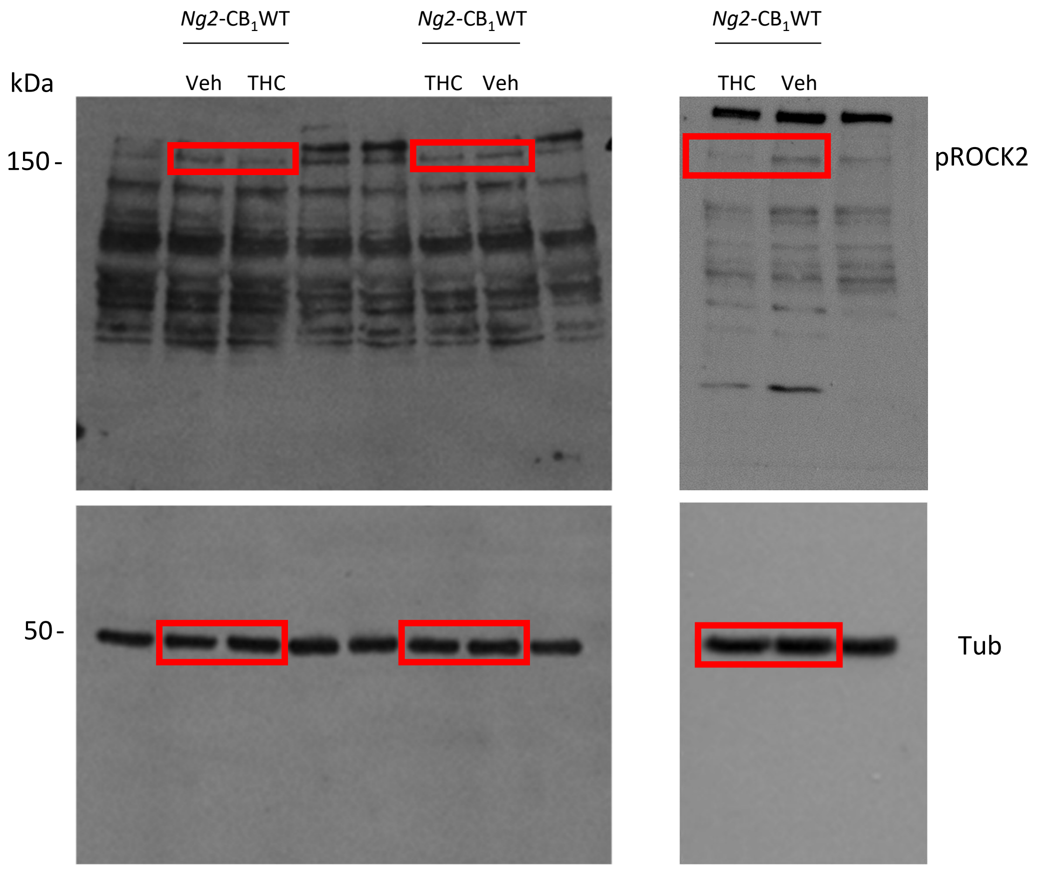

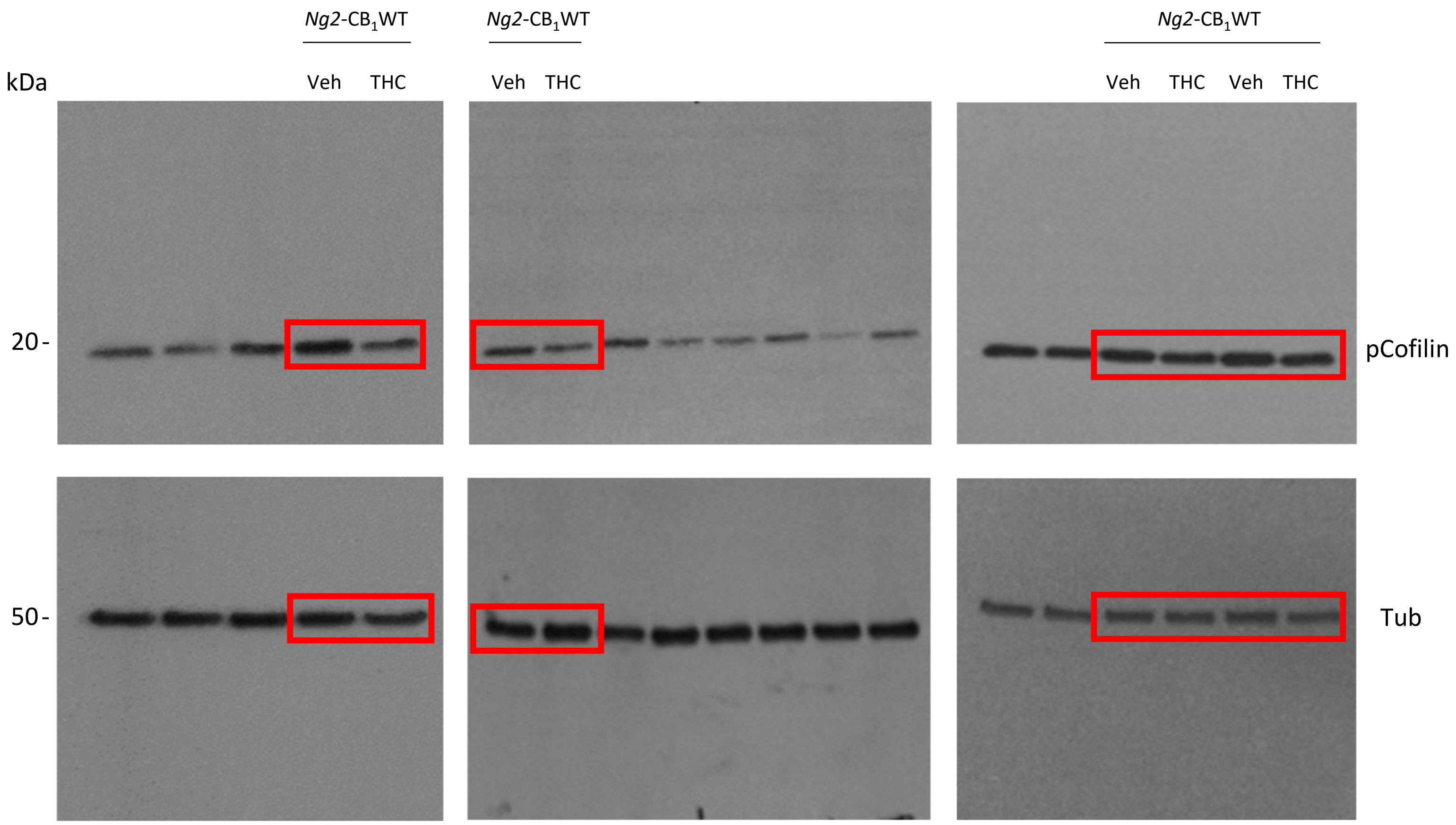

Biological replicates of western blot analysis shown in Figure 4C

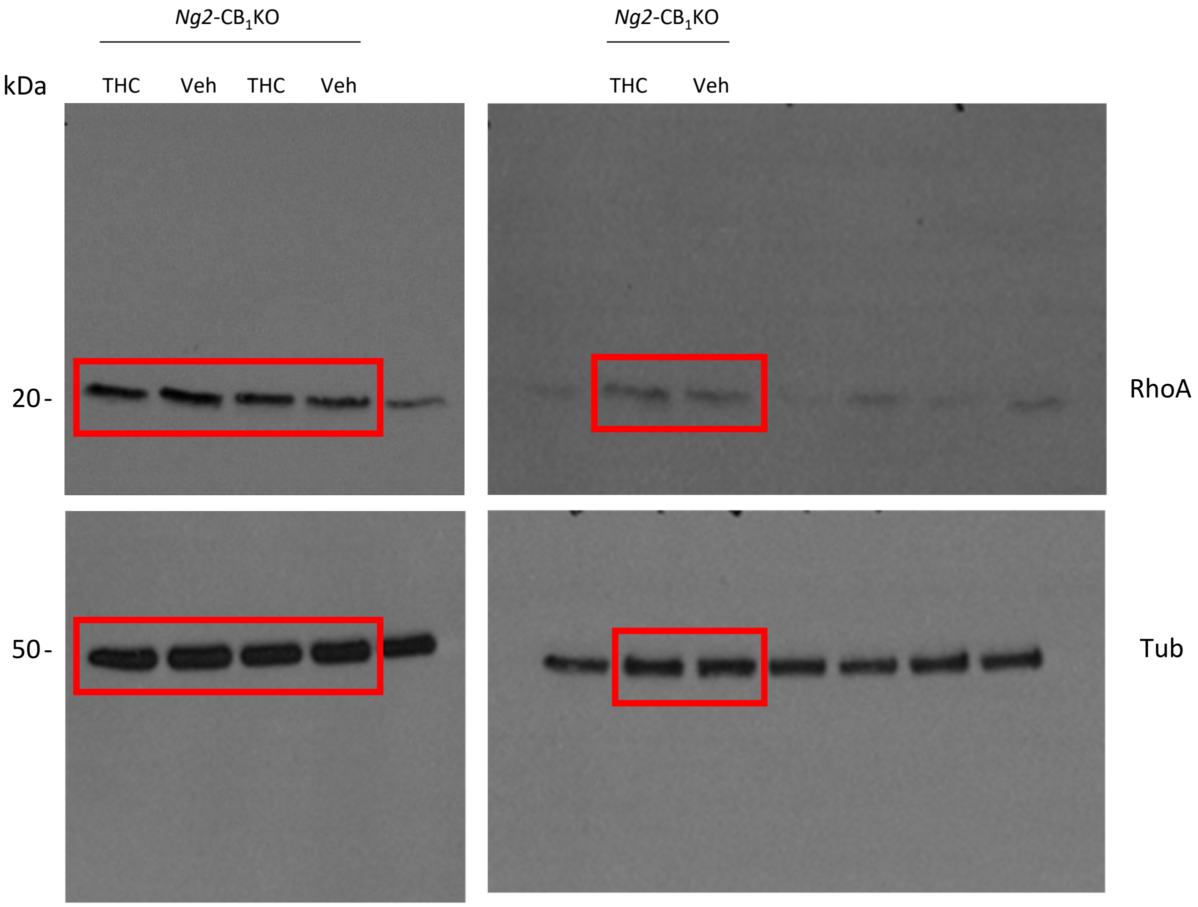

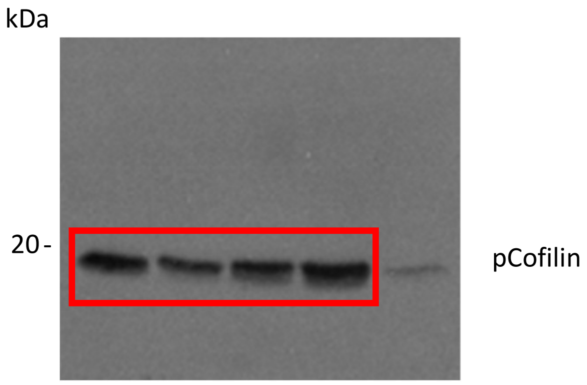

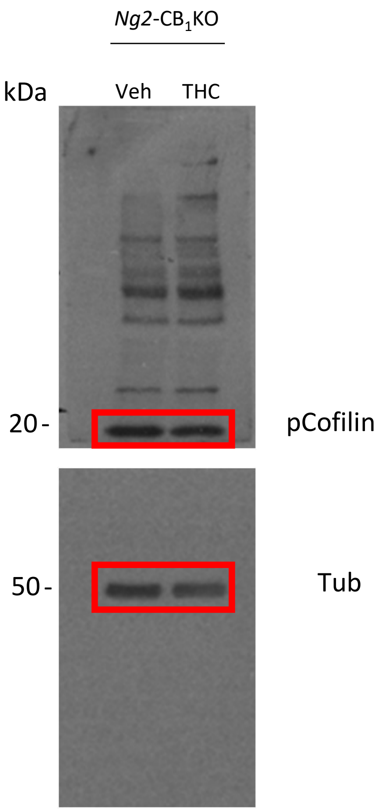

Biological replicates of western blot analysis shown in Figure 4C

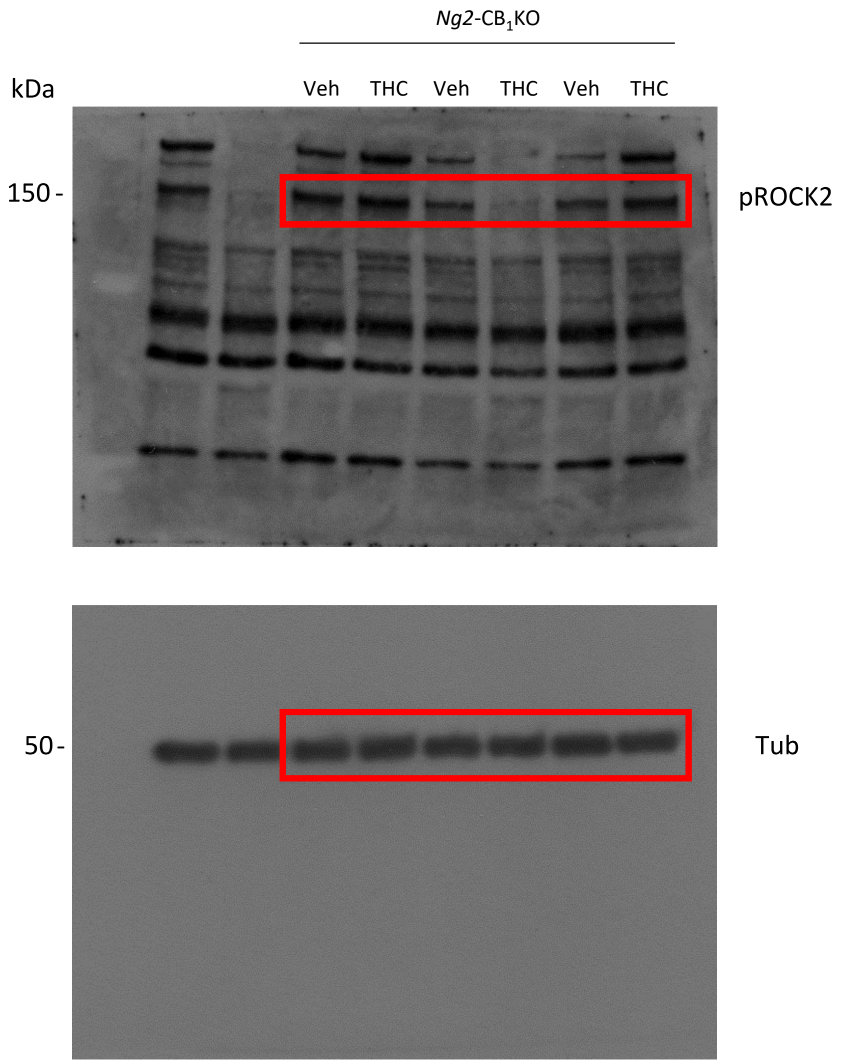

Biological replicates of western blot analysis shown in Figure 4F

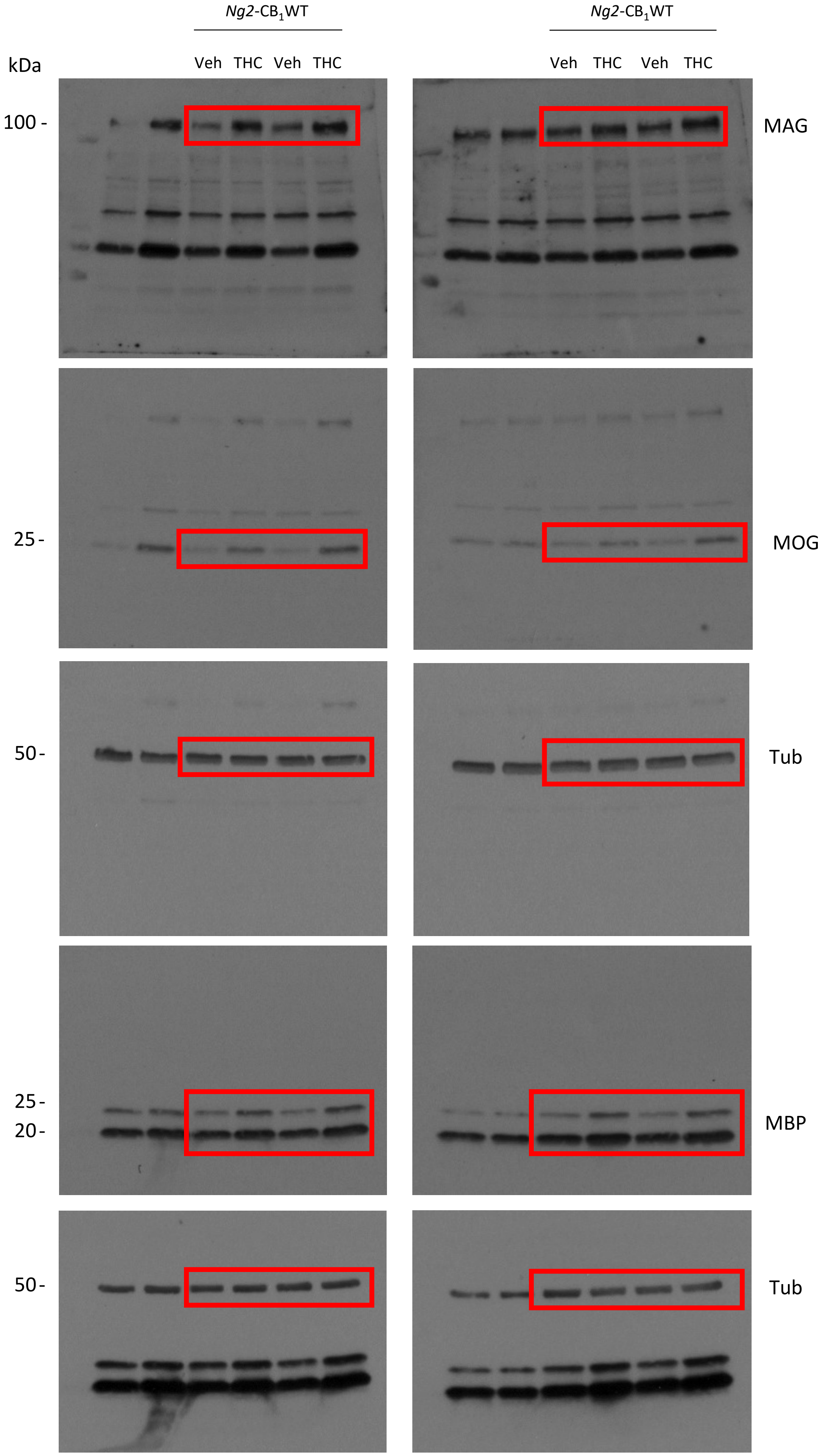

Biological replicates of western blot analysis shown in Figure 4F

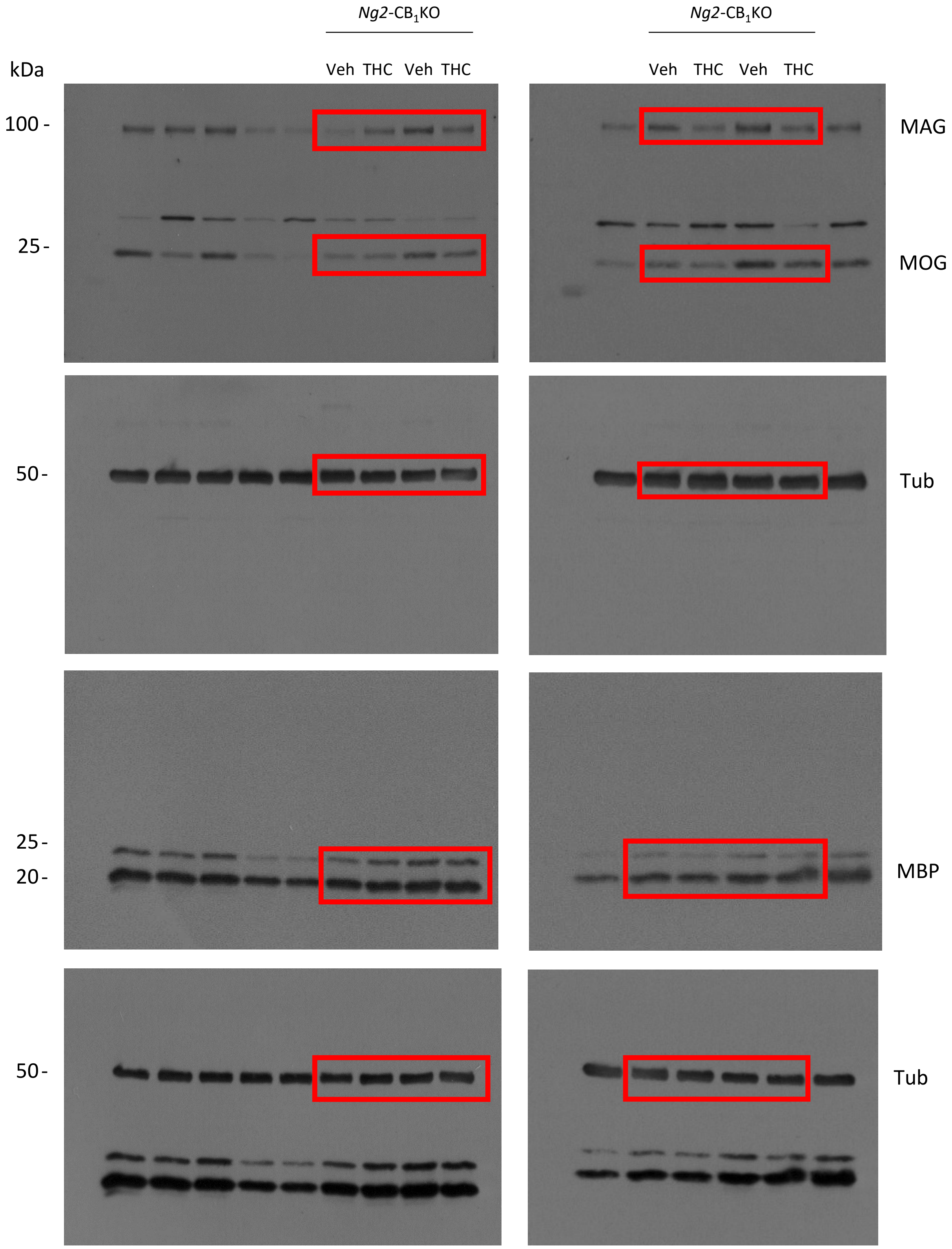

Biological replicates of western blot analysis shown in Figure 5A

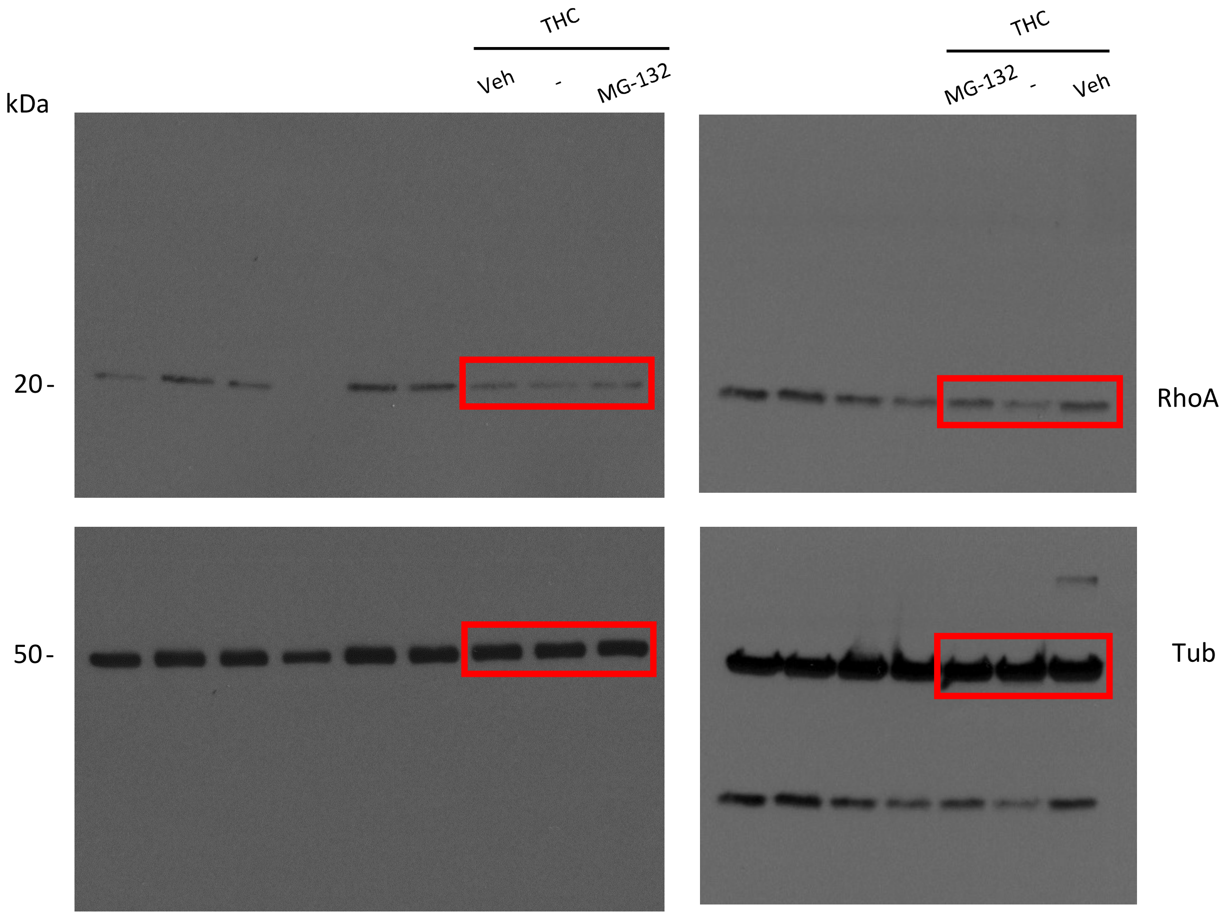

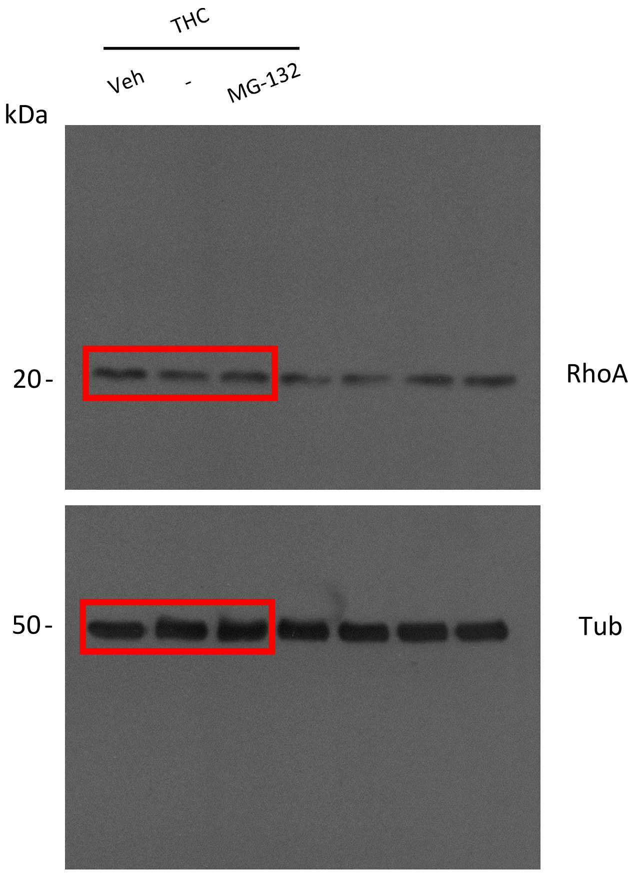

Biological replicates of western blot analysis shown in Figure 6C

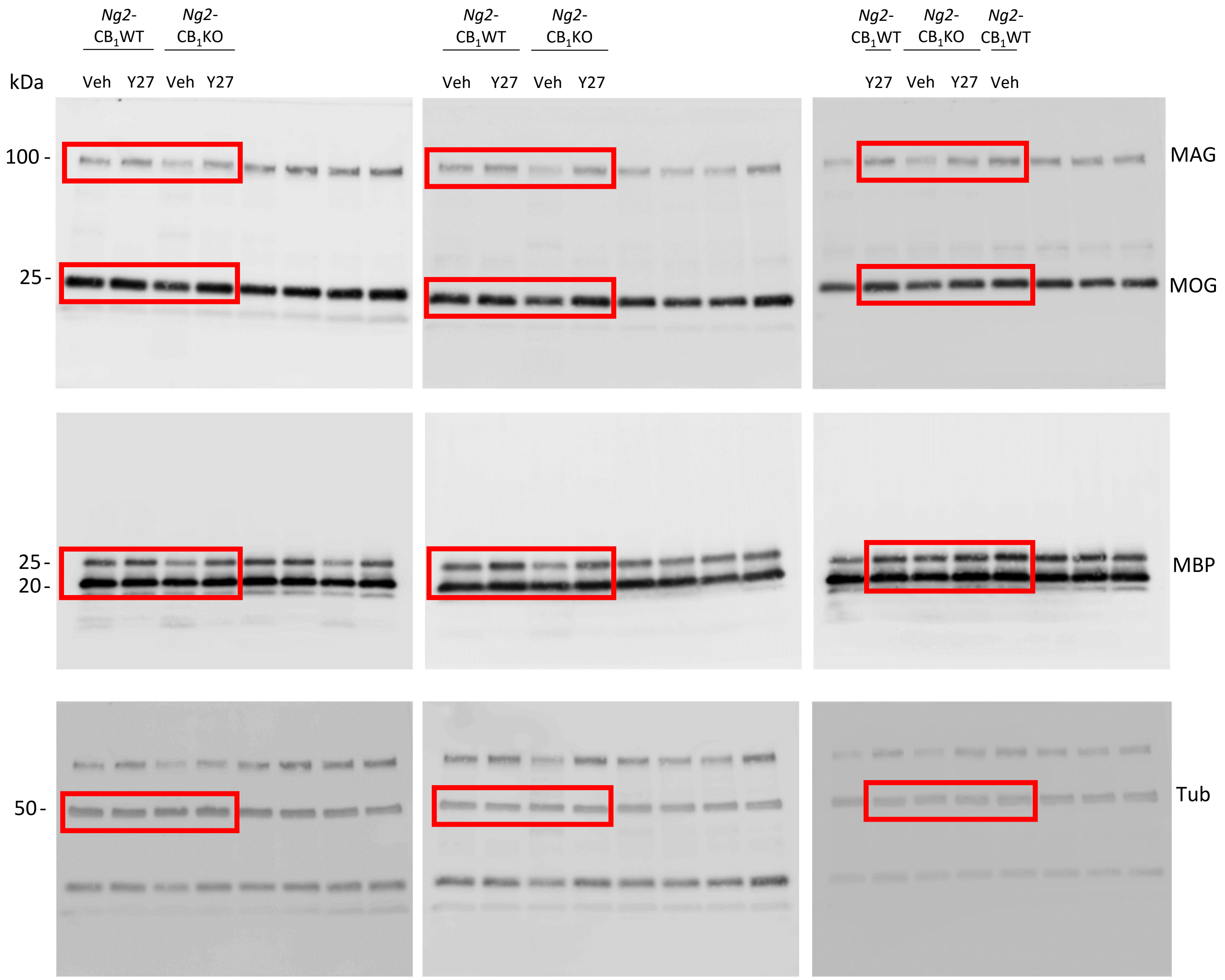

Supplement: Supplementary file 2 — Supplemental information original western blot scans [file 41419_2022_5032_MOESM2_ESM.pdf]
