# Supplementary material for: Cannabinoid CB1 receptor gene inactivation in oligodendrocyte precursors disrupts oligodendrogenesis and myelination in mice
Source: Cell Death Dis. 2022 Jul 7;13(7):585. doi: 10.1038/s41419-022-05032-z (PMC9263142; doi:10.1038/s41419-022-05032-z)
Supplement: Supplementary file 3 — Data sets [file 41419_2022_5032_MOESM3_ESM.docx]

**Data sets for**

Cannabinoid CB_1_ receptor gene inactivation in oligodendrocyte precursors disrupts oligodendrogenesis and myelination in mice

Aníbal Sánchez-de la Torre, Tania Aguado, Alba Huerga-Gómez, Silvia Santamaría, Antonietta Gentile, Juan Carlos Chara, Carlos Matute, Krisztina Monory, Susana Mato, Manuel Guzmán, Beat Lutz, Ismael Galve-Roperh & Javier Palazuelos

Corresponding author: Javier Palazuelos

Email: j.palazuelos@ucm.es

**This PDF file includes:**

Data sets Figures 1 to 6

Data sets Supplementary Figures S1 to S4

**Suplementary Table.**

**Figure 1.**

| **Fig.1B** | **PROTEIN** | **GROUP** | **N number** | **Mean ±S.E.M** | **Shapiro-Wilk test (p-value)** | **Statistics** | **P-value / Significance** |
| --- | --- | --- | --- | --- | --- | --- | --- |
|  | **NG2** | ***Ng*2/Ai6-CB_1_HET** | 4 | 22.58 ± 2.025 | 0.8602 | two-tailed unpaired Student’s t test | <0.0001/ ***; t=9.841 df=6 |
|  |  | ***Ng*2/Ai6-CB_1_KO** | 4 | 44.95 ± 1.035 | 0.9987 |  |  |
|  | **CC1** | ***Ng*2/Ai6-CB_1_HET** | 4 | 77.45 ± 2.043 | 0.8514 | two-tailed unpaired Student’s t test | <0.0001/ ***; t=10.72. df=6 |
|  |  | ***Ng*2/Ai6-CB_1_KO** | 4 | 55.02 ± 0.4592 | 0.4124 |  |  |
|  | **CC1 GSTπ** | ***Ng*2/Ai6-CB_1_HET** | 4 | 44.11 ± 2.207 | 0.885 | two-tailed unpaired Student’s t test | 0.0003/ ***; t=7.602. df=6 |
|  |  | ***Ng*2/Ai6-CB_1_KO** | 4 | 21.75 ± 1.946 | 0.1158 |  |  |
| **Fig.1C** | **PROTEIN/age** | **GROUP** | **N number** | **Mean ±S.E.M** | **Shapiro-Wilk test (p-value)** | **Statistics** | **P-value / Significance** |
| Left panel | **NG2** | ***Ng*2-CB_1_WT** | 6 | 45.17 ± 1.073 | 0.0154 | Mann Whitney test | 0.0043/ ** |
|  |  | ***Ng*2-CB_1_KO** | 5 | 57.7 ± 1.997 | 0.2381 |  |  |
|  | **CC1** | ***Ng*2-CB_1_WT** | 6 | 54.18 ± 1.342 | 0.1225 | two-tailed unpaired Student’s t test | 0.0006/ ***; t=5.140. df=9 |
|  |  | ***Ng*2-CB_1_KO** | 5 | 42.55 ± 1.893 | 0.2413 |  |  |
| Central panel | **P15** | ***Ng*2-CB_1_WT** | 6 | 510.1 ± 43.80 | 0.8523 | Mann Whitney test | 0.0087/** |
|  |  | ***Ng*2-CB_1_KO** | 5 | 354.6 ± 18.38 | 0.0116 |  |  |
|  | **P60** | ***Ng*2-CB_1_WT** | 7 | 950.6 ± 55.41 | 0.4087 | two-tailed unpaired Student’s t test | 0.0003/***; t=4.734. df=14 |
|  |  | ***Ng*2-CB_1_KO** | 9 | 675.4 ± 28.21 | 0.9805 |  |  |
| Right panel | **P15** | ***Ng*2-CB_1_WT** | 6 | 941.2 ± 70.33 | 0.9313 | two-tailed unpaired Student’s t test | 0.2937/n.s.; t=1.115. df=9 |
|  |  | ***Ng*2-CB_1_KO** | 5 | 842.3 ± 47.20 | 0.0852 |  |  |
|  | **P60** | ***Ng*2-CB_1_WT** | 7 | 1053 ± 81.14 | 0.772 | two-tailed unpaired Student’s t test | 0.3216/n.s.; t=1,028. df=14 |
|  |  | ***Ng*2-CB_1_KO** | 9 | 968.2 ± 36.94 | 0.8207 |  |  |
| **Fig.1D** | **PROTEIN** | **GROUP** | **N number** | **Mean ±S.E.M** | **Shapiro-Wilk test (p-value)** | **Statistics** | **P-value / Significance** |
| Left panel | **CC1** | ***Pdgfrα*-CB_1_WT** | 3 | 496.3 ± 52.31 | 0.76 | two-tailed unpaired Student’s t test | 0.0260/*; t=3.452. df=4 |
|  |  | ***Pdgfrα*-CB_1_KO** | 3 | 293.5 ± 26.76 | 0.7239 |  |  |
| Right panel | **NG2** | ***Pdgfrα*-CB_1_WT** | 3 | 43.24 ± 1.863 | 0.5963 | two-tailed unpaired Student’s t test | 0.0092/**; t=4.720. df=4 |
|  |  | ***Pdgfrα*-CB_1_KO** | 3 | 54.57 ± 1.515 | 0.2811 |  |  |
|  | **CC1** | ***Pdgfrα*-CB_1_WT** | 3 | 56.4 ± 1.918 | 0.1773 | two-tailed unpaired Student’s t test | 0.0073/**; t=5.040. df=4 |
|  |  | ***Pdgfrα*-CB_1_KO** | 3 | 45.09 ± 1.164 | 0.1366 |  |  |

| **Fig.1E** | **AGE** | **GROUP** | **N number** | **Mean ±S.E.M** | **Shapiro-Wilk test (p-value)** | **Statistics** | **P-value / Significance** |
| --- | --- | --- | --- | --- | --- | --- | --- |
|  | **P15** | ***Ng*2-CB_1_WT** | 4 | 54.27 ± 4.211 | 0.4031 | two-tailed unpaired Student’s t test | 0.0032/**; t=4.378. df=7 |
|  |  | ***Ng*2-CB_1_KO** | 5 | 29.47 ± 3.784 | 0.6235 |  |  |
|  | **P60** | ***Ng*2-CB_1_WT** | 3 | 64.76 ± 5.955 | 0.2166 | two-tailed unpaired Student’s t test | 0.0328/*; t=3.204. df=4 |
|  |  | ***Ng*2-CB_1_KO** | 3 | 41.43 ± 4.191 | 0.247 |  |  |

**Figure 2.**

| **Fig.2A** | **PROTEIN** | **GROUP** | **N number** | **Mean ±S.E.M** | **Shapiro-Wilk test (p-value)** | **Statistics** | **P-value / Significance** |
| --- | --- | --- | --- | --- | --- | --- | --- |
|  | **MAG** | ***Ng*2-CB_1_WT** | 4 | 1 ± 0.03164 | 0.4731 | two-tailed unpaired Student’s t test | 0.0163/*; t=3.307. df=6 |
|  |  | ***Ng*2-CB_1_KO** | 4 | 0.6037 ± 0.1156 | 0.4675 |  |  |
|  |  | ***Pdgfrα*-CB_1_WT** | 4 | 1 ± 0.1917 | 0.7294 | two-tailed unpaired Student’s t test | 0.0066/**; t=3.809. df=7 |
|  |  | ***Pdgfrα*-CB_1_KO** | 5 | 0.2842 ± 0.07362 | 0.2713 |  |  |
|  | **MOG** | ***Ng*2-CB_1_WT** | 4 | 1 ± 0.08641 | 0.5834 | two-tailed unpaired Student’s t test | 0.0003/***; t=7.345. df=6 |
|  |  | ***Ng*2-CB_1_KO** | 4 | 0.2293 ± 0.05952 | 0.1484 |  |  |
|  |  | ***Pdgfrα*-CB_1_WT** | 4 | 1 ± 0.1431 | 0.5068 | two-tailed unpaired Student’s t test | 0.00597**; t=3.900. df=7 |
|  |  | ***Pdgfrα*-CB_1_KO** | 5 | 0.3755 ± 0.08754 | 0.2126 |  |  |
|  | **MBP** | ***Ng*2-CB_1_WT** | 3 | 1 ± 0.1581 | 0.4326 | two-tailed unpaired Student’s t test | 0.0251/*; t=3.160. df=5 |
|  |  | ***Ng*2-CB_1_KO** | 4 | 0.5422 ± 0.04987 | 0.2573 |  |  |
|  |  | ***Pdgfrα*-CB_1_WT** | 4 | 1 ± 0.05642 | 0.1188 | two-tailed unpaired Student’s t test | 0.0024/**; t=4.629. df=7 |
|  |  | ***Pdgfrα*-CB_1_KO** | 5 | 0.4467 ± 0.09593 | 0.3853 |  |  |
|  |  |  |  |  |  |  |  |
| **Fig.2D** | **GROUP** | **N number** | **Mean ±S.E.M** | **Shapiro-Wilk test (p-value)** | **Statistics** | **P-value / Significance** |  |
|  | ***Ng*2-CB_1_WT** | 3 | 212.9 ± 10.26 | 0.335 | two-tailed unpaired Student’s t test | 0.0445/*; t=2.892. df=4 |  |
|  | ***Ng*2-CB_1_KO** | 3 | 132.7 ± 25.76 | 0.4098 |  |  |  |

| **Fig.2F** | **PROTEIN** | **STRUCTURE** | **GROUP** | **N number** | **Mean ± S.E.M** | **Shapiro-Wilk test (p-value)** | **Statistics** | **P-value / Significance** |
| --- | --- | --- | --- | --- | --- | --- | --- | --- |
|  | **MAG** | **CC** | ***Ng*2-CB_1_WT** | 4 | 1 ± 0.1093 | 0.3155 | two-tailed unpaired Student’s t test | 0.0392/*; t=2.628. df=6 |
|  |  |  | ***Ng*2-CB_1_KO** | 4 | 0.6844 ± 0.04991 | 0.923 |  |  |
|  |  | **HIPP** | ***Ng*2-CB_1_WT** | 3 | 1 ± 0.02973 | 0.0557 | two-tailed unpaired Student’s t test | 0.0395/*; t=3.011. df=4 |
|  |  |  | ***Ng*2-CB_1_KO** | 3 | 0.5849 ± 0.1346 | 0.5079 |  |  |
|  |  | **mPFC** | ***Ng*2-CB_1_WT** | 4 | 1 ± 0.06515 | 0.3368 | two-tailed unpaired Student’s t test | 0.0108/*; t=3.640. df=6 |
|  |  |  | ***Ng*2-CB_1_KO** | 4 | 0.7222 ± 0.03974 | 0.5162 |  |  |
|  |  | **Crb** | ***Ng*2-CB_1_WT** | 4 | 1 ± 0.06092 | 0.3919 | two-tailed unpaired Student’s t test | 0.0021/**; t=5.175. df=6 |
|  |  |  | ***Ng*2-CB_1_KO** | 4 | 0.3954 ± 0.09969 | 0.8845 |  |  |
|  | **MOG** | **CC** | ***Ng*2-CB_1_WT** | 4 | 1 ± 0.09976 | 0.4593 | two-tailed unpaired Student’s t test | 0.0038/**; t=4.561. df=6 |
|  |  |  | ***Ng*2-CB_1_KO** | 4 | 0.4948 ± 0.04814 | 0.5868 |  |  |
|  |  | **HIPP** | ***Ng*2-CB_1_WT** | 3 | 1 ± 0.02676 | 0.4618 | two-tailed unpaired Student’s t test | 0.0175/*; t=3.900. df=4 |
|  |  |  | ***Ng*2-CB_1_KO** | 3 | 0.5412 ± 0.1146 | 0.097 |  |  |
|  |  | **mPFC** | ***Ng*2-CB_1_WT** | 4 | 1 ± 0.04756 | 0.6519 | two-tailed unpaired Student’s t test | 0.0015/**; t=5.539. df=6 |
|  |  |  | ***Ng*2-CB_1_KO** | 4 | 0.6975 ± 0.02686 | 0.2303 |  |  |
|  |  | **Crb** | ***Ng*2-CB_1_WT** | 4 | 1 ± 0.03929 | 0.9004 | two-tailed unpaired Student’s t test | 0.0009/***; t=6.140. df=6 |
|  |  |  | ***Ng*2-CB_1_KO** | 4 | 0.4782 ± 0.07534 | 0.5258 |  |  |
|  | **MBP** | **CC** | ***Ng*2-CB_1_WT** | 4 | 1 ± 0.07123 | 0.7982 | two-tailed unpaired Student’s t test | 0.0005/***; t=6.858. df=6 |
|  |  |  | ***Ng*2-CB_1_KO** | 4 | 0.4769 ± 0.02731 | 0.7694 |  |  |
|  |  | **HIPP** | ***Ng*2-CB_1_WT** | 3 | 1 ± 0.02676 | 0.4618 | two-tailed unpaired Student’s t test | 0.0382/*; t=3.047. df=4 |
|  |  |  | ***Ng*2-CB_1_KO** | 3 | 0.5412 ± 0.1146 | 0.097 |  |  |
|  |  | **mPFC** | ***Ng*2-CB_1_WT** | 4 | 1 ± 0.07413 | 0.3328 | two-tailed unpaired Student’s t test | 0.0159/*; t=3.327. df=6 |
|  |  |  | ***Ng*2-CB_1_KO** | 4 | 0.7324 ± 0.03126 | 0.5723 |  |  |
|  |  | **Crb** | ***Ng*2-CB_1_WT** | 3 | 1 ± 0.1621 | 0.2796 | two-tailed unpaired Student’s t test | 0.0493/*; t=2.582. df=5 |
|  |  |  | ***Ng*2-CB_1_KO** | 4 | 0.6261 ± 0.04282 | 0.8252 |  |  |

| **Fig.2G** | **GROUP** | **N number** | **Mean ± S.E.M** | **Shapiro-Wilk test (p-value)** | **Statistics** | **P-value / Significance** |
| --- | --- | --- | --- | --- | --- | --- |
| Left graph | ***Ng*2-CB_1_WT** | 4 | 396.2 ± 79.3 | 0.781 | two-tailed unpaired Student’s t test | 0.9485/n.s.; t=0.06732. df=6 |
|  | ***Ng*2-CB_1_KO** | 4 | 402.4 ± 45.99 | 0.6888 |  |  |
| Central graph | ***Ng*2-CB_1_WT** | 4 | 3.7 ± 0.03419 | 0.1249 | two-tailed unpaired Student’s t test | <0.0001/***; t=9.205, df=6 |
|  | ***Ng*2-CB_1_KO** | 4 | 2.956 ± 0.07325 | 0.3069 |  |  |
| Right graph | ***Ng*2-CB_1_WT** | 4 | 1.13 ± 0.03808 | 0.2359 | two-tailed unpaired Student’s t test | 0.0071/**; t=3.999, df=6 |
|  | ***Ng*2-CB_1_KO** | 4 | 1.3 ± 0.01903 | 0.2978 |  |  |

**Figure 3.**

|  | | **Test** | **GROUP** | **N number** | **Mean ±S.E.M** | **D'Agostino & Pearson normality test** | **Statistics** | **P-value / Significance** |
| --- | --- | --- | --- | --- | --- | --- | --- | --- |
| **Fig.3A** | Left Graph | **Open Field** | ***Ng*2-CB_1_WT** | 25 | 2359 ± 104 | 0.513 | two-tailed unpaired Student’s t test | 0.0091/**; t=2.737. df=41 |
|  |  |  | ***Ng*2-CB_1_KO** | 18 | 1938 ± 109.5 | 0.4573 |  |  |
|  | Right Graph |  | ***Ng*2-CB_1_WT** | 25 | 5.36 ± 0.5564 | 0.8254 | two-tailed unpaired Student’s t test | 0.0011/**; t=3.502. df=41 |
|  |  |  | ***Ng*2-CB_1_KO** | 18 | 2.722 ± 0.4339 | 0.3857 |  |  |
| **Fig.3B** | Left Graph | **Beam walking** | ***Ng*2-CB_1_WT** | 11 | 0.6364 ± 0.2033 | 0.6505 | two-tailed unpaired Student’s t test | 0.0051/**; t=3.060. df=26 |
|  |  |  | ***Ng*2-CB_1_KO** | 17 | 1.765 ± 0.2647 | 0.9277 |  |  |
|  | Right Graph |  | ***Ng*2-CB_1_WT** | 11 | 6.188 ± 0.2407 | 0.5938 | Mann Whitney test | 0.0016/** |
|  |  |  | ***Ng*2-CB_1_KO** | 17 | 7.835 ± 0.3733 | 0.0247 |  |  |
| **Fig.3C** | Left Graph | **Actitrack** | ***Ng*2-CB_1_WT** | 20 | 1362 ± 40.25 | 0.0695 | two-tailed unpaired Student’s t test | 0.0131/*; t=2.617. df=34 |
|  |  |  | ***Ng*2-CB_1_KO** | 16 | 1175 ± 62,22 | 0.9631 |  |  |
|  | Central Graph |  | ***Ng*2-CB_1_WT** | 20 | 5.159 ± 0.2568 | 0.0057 | Mann Whitney test | 0.0114/* |
|  |  |  | ***Ng*2-CB_1_KO** | 16 | 4.059 ± 0.2721 | 0.8455 |  |  |
|  | Right Graph |  | ***Ng*2-CB_1_WT** | 20 | 1559 ± 73.04 | 0.0022 | Mann Whitney test | 0.0039/** |
|  |  |  | ***Ng*2-CB_1_KO** | 16 | 1214 ± 81.51 | 0.8755 |  |  |
| **Fig.3D** | Left Graph | **EPM** | ***Ng*2-CB_1_WT** | 21 | 17.06 ± 2.644 | 0.1039 | Unpaired t test with Welch's correction | <0.0001/***; t=4.997. df=23.8 |
|  |  |  | ***Ng*2-CB_1_KO** | 14 | 3.214 ± 0.8302 | 0.0686 |  |  |
|  | Right Graph |  | ***Ng*2-CB_1_WT** | 21 | 13.19 ± 2.192 | 0.2938 | Unpaired t test with Welch's correction | 0.0006/***; t=3.909. df=25.6 |
|  |  |  | ***Ng*2-CB_1_KO** | 14 | 4 ± 0.8516 | 0.5251 |  |  |

| **Fig.3E** | Left Graph | **NOR** | ***Ng*2-CB_1_WT** | 24 | 32.55 ± 2.248 | 0.3685 | Unpaired t test with Welch's correction | 0.0107/*; t=2.744. df=27 |
| --- | --- | --- | --- | --- | --- | --- | --- | --- |
|  |  |  | ***Ng*2-CB_1_KO** | 18 | 19.72 ± 4.099 | 0.1847 |  |  |
|  | Right Graph |  | ***Ng*2-CB_1_WT** | 24 | 66.28 ± 1.124 | 0.3685 | Unpaired t test with Welch's correction | 0.0107/*; t=2.744. df=27 |
|  |  |  | ***Ng*2-CB_1_KO** | 18 | 59.86 ± 2.049 | 0.1847 |  |  |
| **Fig.3F** | | **Y-maze** | ***Ng*2-CB_1_WT** | 15 | 33.99 ± 2.615 | 0.6755 | two-tailed unpaired Student’s t test | 0.0007/***; t=3.970. df=21 |
|  |  |  | ***Ng*2-CB_1_KO** | 8 | 16.44 ± 3.546 | 0.6758 |  |  |

**Figure 4.**

| **Fig.4A** | **PROTEIN** | **GROUP** | **N number** | **Mean ±S.E.M** | **Shapiro-Wilk test (p-value)** | **Statistics** | **P-value / Significance** |
| --- | --- | --- | --- | --- | --- | --- | --- |
|  | **RhoA** | ***Ng*2-CB_1_WT** | 3 | 1 ± 0.06198 | 0.1391 | two-tailed unpaired Student’s t test | 0.0156/*; t=4.040. df=4 |
|  |  | ***Ng*2-CB_1_KO** | 3 | 1.497 ± 0.1062 | 0.7761 |  |  |
|  | **pROCK2** | ***Ng*2-CB_1_WT** | 4 | 1 ± 0.07059 | 0.8283 | two-tailed unpaired Student’s t test | 0.0071/**; t=3.996. df=6 |
|  |  | ***Ng*2-CB_1_KO** | 4 | 1.713 ± 0.1639 | 0.4883 |  |  |
|  | **pCofilin** | ***Ng*2-CB_1_WT** | 4 | 1 ± 0.03188 | 0.9878 | two-tailed unpaired Student’s t test | 0.0045/**; t=4.419. df=6 |
|  |  | ***Ng*2-CB_1_KO** | 4 | 1.641 ± 0.1414 | 0.3703 |  |  |

| **Fig4.C** | **PROTEIN** | **GROUP** | **N number** | **Mean ± S.E.M** | **Shapiro-Wilk test (p-value)** | **Statistics** | **P-value / Significance** |
| --- | --- | --- | --- | --- | --- | --- | --- |
|  | **RhoA** | ***Ng2*-CB_1_WT-Veh** | 4 | 1 ± 0.0662 | 0.6637 | two-tailed unpaired Student’s t test | 0.0021/**; t=5.146. df=6 |
|  |  | ***Ng2*-CB_1_WT-THC** | 4 | 0.4699 ± 0.07893 | 0.7607 |  |  |
|  | **pROCK2** | ***Ng2*-CB_1_WT-Veh** | 3 | 1 ± 0.1273 | 0.3631 | two-tailed unpaired Student’s t test | 0.0097/**; t=4.651. df=4 |
|  |  | ***Ng2*-CB_1_WT-THC** | 3 | 0.3922 ± 0.02959 | 0.1866 |  |  |
|  | **pCofilin** | ***Ng2*-CB_1_WT-Veh** | 4 | 1 ± 0.07321 | 0.1328 | two-tailed unpaired Student’s t test | 0.0129/*; t=3.498. df=6 |
|  |  | ***Ng2*-CB_1_WT-THC** | 4 | 0.558 ± 0.1112 | 0.4812 |  |  |
|  | **RhoA** | ***Ng2*-CB_1_KO-Veh** | 3 | 1 ± 0.07465 | 0.9891 | two-tailed unpaired Student’s t test | 0.54798/ n.s.; t=0.6555. df=4 |
|  |  | ***Ng2*-CB_1_KO-THC** | 3 | 0.9228 ± 0.09114 | 0.1391 |  |  |
|  | **pROCK2** | ***Ng2*-CB_1_KO-Veh** | 3 | 1 ± 0.208 | 0.7618 | two-tailed unpaired Student’s t test | 0.4739/n.s.; t=0.7896. df=4 |
|  |  | ***Ng2*-CB_1_KO-THC** | 3 | 0.8032 ± 0.1373 | 0.7412 |  |  |
|  | **pCofilin** | ***Ng2*-CB_1_KO-Veh** | 3 | 1 ± 0.2363 | 0.03 | Mann Whitney test | >0.9999/n.s. |
|  |  | ***Ng2*-CB_1_KO-THC** | 3 | 1.035 ± 0.1136 | 0.1288 |  |  |

| **Fig.4E** | **GROUP** | **N number** | **Mean ±S.E.M** | **ShapiroWilk test (p-value)** | **Statistics** | **Tukey's multiple comparisons P-value / Significance** |
| --- | --- | --- | --- | --- | --- | --- |
|  | ***Ng2*/Ai6-CB_1_HET-Veh** | 3 | 32.71 ± 1.867 | 0.2718 | Two-way  ANOVA | Ng2/Ai6-CB1HET-Veh vs Ng2/Ai6-CB1HET-THC: P<0.0001/*** |
|  | ***Ng2*/Ai6-CB_1_HET-THC** | 3 | 52.23 ± 1.182 | 0.3929 |  | Ng2/Ai6-CB1HET-Veh vs Ng2/Ai6-CB1KO-Veh: P=0.0001/*** |
|  | ***Ng2*/Ai6-CB_1_KO-Veh** | 3 | 17.14 ± 0.7197 | 0.4681 |  | Ng2/Ai6-CB1HET-Veh vs Ng2/Ai6-CB1KO-THC: P=0.0003/*** |
|  | ***Ng2*/Ai6-CB_1_KO-THC** | 3 | 18.95 ± 0.9668 | 0.2594 |  | Ng2/Ai6-CB1KO-Veh vs Ng2/Ai6-CB1KO-THC: P= 0.7447/n.s. |

| **Fig.4F** | **PROTEIN** | **GROUP** | **N number** | **Mean ±S.E.M** | **Shapiro-Wilk test (p-value)** | **Statistics** | **P-value / Significance** |
| --- | --- | --- | --- | --- | --- | --- | --- |
|  | **MAG** | ***Ng2*-CB_1_WT-Veh** | 4 | 1 ± 0.1398 | 0.6009 | two-tailed unpaired Student’s t test | 0.0104/*; t=3.677. df=6 |
|  |  | ***Ng2*-CB_1_WT-THC** | 4 | 2.77 ± 0.4606 | 0.9964 |  |  |
|  | **MOG** | ***Ng2*-CB_1_WT-Veh** | 4 | 1 ± 0.0427 | 0.3843 | two-tailed unpaired Student’s t test | 0.0001/***; t=8.815. df=6 |
|  |  | ***Ng2*-CB_1_WT-THC** | 4 | 2.239 ± 0.2677 | 0.1179 |  |  |
|  | **MBP** | ***Ng2*-CB_1_WT-Veh** | 4 | 1 ± 0.06306 | 0.255 | two-tailed unpaired Student’s t test | 0.0013/**; t=5.643. df=6 |
|  |  | ***Ng2*-CB_1_WT-THC** | 4 | 1.474 ± 0.05545 | 0.0832 |  |  |
|  | **MAG** | ***Ng2*-CB_1_KO-Veh** | 4 | 1 ± 0.2847 | 0.3147 | two-tailed unpaired Student’s t test | 0.4133/n.s.; t=0.8787. df=6 |
|  |  | ***Ng2*-CB_1_KO-THC** | 4 | 0.7095 ± 0.168 | 0.2374 |  |  |
|  | **MOG** | ***Ng2*-CB_1_KO-Veh** | 4 | 1 ± 0.3661 | 0.6744 | Mann Whitney test | 0.6857/n.s. |
|  |  | ***Ng2*-CB_1_KO-THC** | 4 | 0.7235 ± 0.1885 | 0.0346 |  |  |
|  | **MBP** | ***Ng2*-CB_1_KO-Veh** | 4 | 1 ± 0.146 | 0.2964 | two-tailed unpaired Student’s t test | 0.9261/n.s.; t=0.09672. df=6 |
|  |  | ***Ng2*-CB_1_KO-THC** | 4 | 0.9754 ± 0.2082 | 0.8721 |  |  |

**Figure 5.**

| **Fig.5A** | **PROTEIN** | **GROUP** | **N number** | **Mean ± S.E.M** | **Shapiro-Wilk test (p-value)** | | | **Statistics** | **Interaction** | | **Tukey's multiple comparisons P-value / Significance** | |
| --- | --- | --- | --- | --- | --- | --- | --- | --- | --- | --- | --- | --- |
|  | **RhoA** | ***Ng2*-CB_1_WT-Veh** | 3 | 1 ± 0.08755 | 0.6073 | | | One-way  ANOVA | F (2. 6) = 5.141 | | *Ng2*-CB_1_WT-Veh vs *Ng2*-CB_1_WT-THC: P=0.0423/* | |
|  |  | ***Ng2*-CB_1_WT-THC** | 3 | 0.5455 ± 0.09148 | 0.386 | | |  |  |  | *Ng2*-CB_1_WT-Veh vs *Ng2*-CB_1_WT-THC+MG132: P=0.2937/n.s. | |
|  |  | ***Ng2*-CB_1_WT-THC+MG132** | 3 | 0.7646 ± 0.1188 | 0.3126 | | |  | P=0.049/* | | *Ng2*-CB_1_WT-THC vs *Ng2*-CB_1_WT-THC+MG132: P=0.3372/n.s. | |
| **Fig.5B** | **GROUP** | **N number** | **Mean ±S.E.M** | **Shapiro-Wilk test (p-value)** | | **Statistics** | **Interaction** | | | **Tukey's multiple comparisons P-value / Significance** | |  |
|  | ***Ng2-*CB_1_WT-Veh** | 3 | 34.01 ± 3.903 | 0.9602 | | One-way  ANOVA | F (2. 7) = 48.81 | | | *Ng2*-CB_1_WT-Veh vs *Ng2*-CB_1_WT-THC: P=0.0002/*** | |  |
|  | ***Ng2*-CB_1_WT-THC** | 3 | 65.97 ± 0.8741 | 0.9878 | |  |  |  |  | *Ng2*-CB_1_WT-Veh vs *Ng2*-CB_1_WT-THC+MG132:P= 0.9482/n.s. | |  |
|  | ***Ng2*-CB_1_WT-THC+MG132** | 4 | 32.87 ± 2.315 | 0.4852 | |  | P <0.0001/*** | | | *Ng2*-CB_1_WT-THC vs *Ng2*-CB_1_WT-THC+MG132: P= 0.0001/*** | |  |

| **Fig.5C** | **RhoA** | **GROUP** | **N number** | **Mean ±S.E.M** | **Shapiro-Wilk test (p-value)** | **Statistics** | **P-value / Significance** |
| --- | --- | --- | --- | --- | --- | --- | --- |
|  | **PROTEIN** | ***Ng2*-CB_1_WT-Veh** | 4 | 1 ± 0.02972 | 0.9948 | two-tailed unpaired Student’s t test | 0.0016/**; t=5.004. df=7 |
|  |  | ***Ng2*-CB_1_WT-THC** | 5 | 0.744 ± 0.03881 | 0.2956 |  |  |
|  | **ACTIVITY** | ***Ng2*-CB_1_WT-Veh** | 4 | 1 ± 0.00962 | 0.7509 | two-tailed unpaired Student’s t test | 0.0002/***; t=8.078. df=6 |
|  |  | ***Ng2*-CB_1_WT-THC** | 4 | 0.7011 ± 0.03573 | 0.1846 |  |  |
|  | **mRNA** | ***Ng2*-CB_1_WT-Veh** | 3 | 1 ± 0.06281 | 0.9763 | two-tailed unpaired Student’s t test | 0.727/n.s.; t=0.3746. df=4 |
|  |  | ***Ng2*-CB_1_WT-THC** | 3 | 1.031 ± 0.06068 | 0.305 |  |  |

| **Fig.5D** | **GROUP** | **N number** | **Mean ±S.E.M** | **Shapiro-Wilk test (p-value)** | **Statistics** | **P-value / Significance** |
| --- | --- | --- | --- | --- | --- | --- |
|  | **VEH** | 3 | 55.58 ± 3.586 | 0.8741 | two-tailed unpaired Student’s t test | 0.009/**; t=4.741. df=4 |
|  | **THC** | 3 | 73.7 ± 1.326 | 0.3716 |  |  |

| **Fig.5E** | **GROUP** | **N number** | **Mean ±S.E.M** | **Shapiro-Wilk test (p-value)** | **Statistics** | **Interaction** | **Dunn´s test** |
| --- | --- | --- | --- | --- | --- | --- | --- |
|  | **SiC-Veh** | 7 | 14.93± 3.778 | 0.5774 | One-way ANOVA Kruskal-Wallis | P=0.0474/* | SiC-Veh vs SiC-THC: P=0.0445/* |
|  | **SiC-THC** | 9 | 49.65 ± 11.84 | 0.5252 |  |  | SiC-THC vs SiCul3-THC: P=0.037/* |
|  | **SiCul3-THC** | 7 | 19.52 ± 10.55 | 0.0152 |  |  | SiC-THC vs SiSmurf1-THC: P=0.012/* |
|  | **SiSmurf1-THC** | 5 | 8.889 ± 6.479 | 0.0214 |  |  | SiC-THC vs SiSCF-THC: P=0.8032/n.s. |
|  | **SiSCF-THC** | 4 | 36.07 ± 8.593 | 0.8635 |  |  |  |

**Figure 6.**

| **Fig.6B** | **GROUP** | **N number** | **Mean ±S.E.M** | **Shapiro-Wilk normality test** | **Statistics** | **Tukey's multiple comparisons P-value / Significance** |
| --- | --- | --- | --- | --- | --- | --- |
|  | ***Ng2*/Ai6-CB_1_HET-Veh** | 8 | 55.43 ± 2.117 | 0.171 | Two-way  ANOVA | *Ng2*/Ai6-CB_1_HET-Veh vs *Ng2*/Ai6-CB_1_HET-Y27632: P=0.6673/n.s. |
|  | ***Ng2*/Ai6-CB_1_HET-Y27632** | 8 | 58.85 ± 1.735 | 0.8761 |  |  |
|  | ***Ng2*/Ai6-CB_1_KO-Veh** | 6 | 43.49 ± 2.497 | 0.5689 |  | *Ng2*/Ai6-CB_1_HET-Veh vs *Ng2*/Ai6-CB_1_KO-Veh: P= 0.0051/** |
|  | ***Ng2*/Ai6-CB_1_KO-Y27632** | 9 | 55.76 ± 2.231 | 0.1902 |  | *Ng2*/Ai6-CB_1_KO-Veh vs; *Ng2*/Ai6-CB_1_KO-Y27632; P= 0.0031/** |

| **Fig.6C** | **PROTEIN** | **GROUP** | **N number** | **Mean ±S.E.M** | **Shapiro-Wilk normality test** | **Statistics** | **Tukey's multiple comparisons P-value / Significance** |
| --- | --- | --- | --- | --- | --- | --- | --- |
|  | **MAG** | ***Ng*2-CB_1_WT-Veh** | 3 | 1 ± 0.1018 | 0.6881 | Two-way  ANOVA | *Ng2*-CB_1_WT-Veh vs. *Ng2*-CB_1_WT-Y27632: P= 0.4107/n.s. |
|  |  | ***Ng*2-CB_1_WT-Y27632** | 3 | 1.2 ± 0.09507 | 0.0513 |  | *Ng2*-CB_1_WT-Veh vs. *Ng2*-CB_1_KO-Veh: P=0.0494/* |
|  |  | ***Ng*2-CB_1_KO-Veh** | 3 | 0.6075 ± 0.0271 | 0.2244 |  | *Ng2*-CB_1_KO-Veh vs. *Ng2*-CB_1_KO-Y27632: P=0.0203/* |
|  |  | ***Ng*2-CB_1_KO-Y27632** | 3 | 1.079 ± 0.09871 | 0.9284 |  |  |
|  | **MOG** | ***Ng*2-CB_1_WT-Veh** | 3 | 1 ± 0.03456 | 0.2971 | Two-way  ANOVA | *Ng2*-CB_1_WT-Veh vs. *Ng2*-CB_1_WT-Y27632: P= 0.5215/n.s. |
|  |  | ***Ng*2-CB_1_WT-Y27632** | 3 | 1.111 ± 0.04393 | 0.4016 |  | *Ng2*-CB1WT-Veh vs. *Ng2*-CB1KO-Veh: P=0.0385/* |
|  |  | ***Ng*2-CB_1_KO-Veh** | 3 | 0.7358 ± 0.04385 | 0.2377 |  | *Ng2*-CB_1_KO-Veh vs. *Ng2*-CB_1_KO-Y27632: P=0.0234/* |
|  |  | ***Ng*2-CB_1_KO-Y27632** | 3 | 1.028 ± 0.08427 | 0.2561 |  |  |
|  | **MBP** | ***Ng*2-CB_1_WT-Veh** | 3 | 1 ± 0.0724 | 0.3826 | Two-way  ANOVA | *Ng2*-CB_1_WT-Veh vs. *Ng2*-CB_1_WT-Y27632: P= 0.9813/n.s. |
|  |  | ***Ng*2-CB_1_WT-Y27632** | 3 | 1.034 ± 0.08336 | 0.8448 |  | *Ng2*-CB_1_WT-Veh vs. *Ng2*-CB_1_KO-Veh: P=0.0461/* |
|  |  | ***Ng*2-CB_1_KO-Veh** | 3 | 0.7011 ± 0.02382 | 0.2717 |  | *Ng2*-CB_1_KO-Veh vs. *Ng2*-CB_1_KO-Y27632: P=0.0312/* |
|  |  | ***Ng*2-CB_1_KO-Y27632** | 3 | 1.026 ± 0.06365 | 0.6896 |  |  |

|  | **GROUP** | **N number** | **Mean ±S.E.M** | **D'Agostino & Pearson normality test** | **Statistics** | **Tukey's multiple comparisons P-value / Significance** |
| --- | --- | --- | --- | --- | --- | --- |
| **Fig.6D** | ***Ng*2-CB_1_WT-Veh** | 17 | 3286 ± 139.8 | 0.539 | Two-way  ANOVA | *Ng2*-CB_1_WT-Veh vs. *Ng2*-CB_1_WT-Y27632: P= 0.91/n.s. |
|  | ***Ng*2-CB_1_WT-Y27632** | 18 | 3152 ± 135.8 | 0.3271 |  | *Ng2*-CB_1_WT-Veh vs. *Ng2*-CB_1_KO-Veh: P=0.0015/** |
|  | ***Ng*2-CB_1_KO-Veh** | 21 | 2543 ± 129.6 | 0.3478 |  | *Ng2*-CB_1_KO-Veh vs. *Ng2*-CB_1_KO-Y27632: P=0.0429/* |
|  | ***Ng*2-CB_1_KO-Y27632** | 23 | 3025 ± 129.4 | 0.9103 |  |  |
| **Fig.6E** | ***Ng*2-CB_1_WT-Veh** | 17 | 0.8824 ± 0.1895 | 0.2575 | Two-way  ANOVA | *Ng2*-CB_1_WT-Veh vs. *Ng2*-CB_1_WT-Y27632: P= 0.4004/n.s. |
|  | ***Ng*2-CB_1_WT-Y27632** | 21 | 0.4762 ±0.1313 | 0.2253 |  | *Ng2*-CB_1_WT-Veh vs. *Ng2*-CB_1_KO-Veh: P=0.0345/* |
|  | ***Ng*2-CB_1_KO-Veh** | 22 | 1.591 ± 0.1821 | 0.5047 |  | *Ng2*-CB_1_KO-Veh vs. *Ng2*-CB_1_KO-Y27632: P=0.0164/* |
|  | ***Ng*2-CB_1_KO-Y27632** | 22 | 0.8636 ± 0.1895 | 0.3155 |  |  |
| **Fig.6F** | ***Ng*2-CB_1_WT-Veh** | 18 | 44.47 ± 3.666 | 0.8555 | Two-way  ANOVA | *Ng2*-CB_1_WT-Veh vs. *Ng2*-CB_1_WT-Y27632: P= 0.9994/n.s. |
|  | ***Ng*2-CB_1_WT-Y27632** | 18 | 45.22 ± 5.343 | 0.5131 |  | *Ng2*-CB_1_WT-Veh vs. *Ng2*-CB_1_KO-Veh: P=0.0116/* |
|  | ***Ng*2-CB_1_KO-Veh** | 21 | 25.62 ± 3.616 | 0.0993 |  | *Ng2*-CB_1_KO-Veh vs. *Ng2*-CB_1_KO-Y27632: P=0.0313/* |
|  | ***Ng*2-CB_1_KO-Y27632** | 22 | 41.51 ± 4.003 | 0.25 |  |  |
| **Fig.6G** | ***Ng*2-CB_1_WT-Veh** | 16 | 39.29 ± 5.478 | 0.0118 | Two-way  ANOVA | *Ng2*-CB_1_WT-Veh vs. *Ng2*-CB_1_WT-Y27632: P= 0.9551/n.s. |
|  | ***Ng*2-CB_1_WT-Y27632** | 19 | 43.32 ± 6.364 | 0.5041 |  | *Ng2*-CB_1_WT-Veh vs. *Ng2*-CB_1_KO-Veh: P=0.0481/* |
|  | ***Ng*2-CB_1_KO-Veh** | 22 | 19.28 ± 4.464 | 0.3413 |  | *Ng2*-CB_1_KO-Veh vs. *Ng2*-CB_1_KO-Y27632: P=0.0406/* |
|  | ***Ng*2-CB_1_KO-Y27632** | 24 | 37.71 ± 4.397 | 0.8952 |  |  |

**Supplementary Figure 1.**

| **PROTEIN** | **GROUP** | **N number** | **Mean ±S.E.M** | **Shapiro-Wilk test (p-value)** | **Statistics** | **P-value / Significance** |
| --- | --- | --- | --- | --- | --- | --- |
| **Olig2** | ***Ng*2/Ai6-CB_1_HET** | 4 | 78.79 ± 2.287 | 0.1346 | two-tailed unpaired Student’s t test | 0.4557/n.s.; t=0.7972, df=6 |
|  | ***Ng*2/Ai6-CB_1_KO** | 4 | 76.58 ± 1.583 | 0.859 |  |  |

**Supplementary Figure 2.**

| **PROTEIN** | **GROUP** | **N number** | **Mean ±S.E.M** | **Shapiro-Wilk test (p-value)** | **Statistics** | **P-value / Significance** |
| --- | --- | --- | --- | --- | --- | --- |
| **NG2** | ***Ng*2-CB_1_WT** | 8 | 14.48 ± 2.744 | 0.6024 | two-tailed unpaired Student’s t test | <0.0001/***; t=6.648. df=14 |
|  | ***Ng*2-CB_1_KO** | 8 | 34.69 ± 1.309 | 0.1596 |  |  |
| **CC1** | ***Ng*2-CB_1_WT** | 8 | 85.5 ± 2.791 | 0.6651 | two-tailed unpaired Student’s t test | <0.0001/***; t=6.618. df=14 |
|  | ***Ng*2-CB_1_KO** | 8 | 64.96 ± 1.357 | 0.0666 |  |  |

**Supplementary Figure 3.**

| **AGE** | **GROUP** | **N number** | **Mean ±S.E.M** | **Shapiro-Wilk test (p-value)** | **Statistics** | **P-value / Significance** |
| --- | --- | --- | --- | --- | --- | --- |
| **P15** | ***Ng*2-CB_1_WT** | 4 | 305.6 ± 31.58 | 0.1486 | two-tailed unpaired Student’s t test | 0.0003/***; t=7.602. df=6 |
|  | ***Ng*2-CB_1_KO** | 5 | 93.62 ± 13.6 | 0.6982 |  |  |
| **P60** | ***Ng*2-CB_1_WT** | 3 | 619.4 ± 89.15 | 0.5836 | two-tailed unpaired Student’s t test | 0.0315/*; t=3.245. df=4 |
|  | ***Ng*2-CB_1_KO** | 3 | 295.4 ± 44.95 | 0.7835 |  |  |

**Supplementary Figure 4.**

| **GENE** | **GROUP** | **N number** | **Mean ± S.E.M** | **Shapiro-Wilk test (p-value)** | **Statistics** | **P-value / Significance** |
| --- | --- | --- | --- | --- | --- | --- |
| **MAG** | ***Ng*2-CB_1_WT** | 4 | 1 ± 0.05333 | 0.9661 | two-tailed unpaired Student’s t test | 0.0016/**; t=5.459. df=6 |
|  | ***Ng*2-CB_1_KO** | 4 | 0.56745 ± 0.05863 | 0.6862 |  |  |
| **MOG** | ***Ng*2-CB_1_WT** | 4 | 1 ± 0.07788 | 0.7436 | two-tailed unpaired Student’s t test | 0.00777/**; t=3.926. df=6 |
|  | ***Ng*2-CB_1_KO** | 4 | 0.6598 ± 0.038 | 0.2629 |  |  |
| **MBP** | ***Ng*2-CB_1_WT** | 4 | 1 ± 0.06531 | 0.9768 | two-tailed unpaired Student’s t test | 0.0006/***; t=6.637. df=6 |
|  | ***Ng*2-CB_1_KO** | 4 | 0.5278 ± 0.02821 | 0.0645 |  |  |
